# Going within, between and beyond: An exploration of regular Ashtanga Yoga practitioners’ conceptualizations of five dimensions of wellbeing

**DOI:** 10.3389/fpsyg.2022.1018620

**Published:** 2022-12-21

**Authors:** Daniela Ramirez-Duran, Helen Stokes, Margaret L. Kern

**Affiliations:** ^1^Centre for Wellbeing Science, Melbourne Graduate School of Education, University of Melbourne, Melbourne, VIC, Australia; ^2^Melbourne Graduate School of Education, University of Melbourne, Melbourne, VIC, Australia

**Keywords:** dimensions of wellbeing, Ashtanga Yoga, reflexive thematic analysis, self, meta-awareness, equanimity

## Abstract

Yoga is an embodied practice underpinned by philosophical elements, seeking to evolve different dimensions of human existence for optimal functioning in relation to oneself, others and beyond. This mixed-methods research focused on 137 regular Ashtanga Yoga practitioners (AYPs) by investigating their conceptualizations of five dimensions of wellbeing (i.e., physical, emotional, psychological, social, spiritual). Conceptualizations were analysed through word count analysis and Reflexive Thematic Analysis separately for each dimension, yielding four themes in each case, which partly aligned with existing wellbeing and yoga models, and partly extended on the existing literature. Further higher level analysis identified shared meanings across these five dimensions, expressed in themes grouped within five topics (i.e., freedom from and managing suffering, a positive and integrated sense of self, a sense of equanimity and steadiness, the self in relation to others and the world, meta-awareness). Furthermore, it also portrayed each dimension as multileveled, represented in three levels of human functioning. The *foundational level* encompassed the absence and managing suffering, representing functioning in coping and recovery mode. The *optimal functioning level* included a positive and integrated sense of self, a sense of equanimity and steadiness, and the self in relation to others and the world, representing functioning in personal development and growth mode. The *contemplative and transcendental level* involved meta-awareness in every dimension of wellbeing, representing functioning in transpersonal mode. As a result, we propose a preliminary model informed both by this empirical work and previous theories. While the continuousness of themes across dimensions reinforces the importance of embodiment and transcendence in wellbeing frameworks, the notion of the self as a multi-level system could be further explored in relation to knowing about and cultivating wellbeing.

## Introduction

As wellbeing science continues to expand, the vast diversity of theoretical frameworks and self-report measurements on individual subjective wellbeing has paved multiple avenues to understand and measure it (Linton et al., 2016). However, this can pose a significant challenge when choosing what and how to measure wellbeing in particular contexts. Adhering to a specific framework and measurement may lead to a partial and narrow understanding of what wellbeing means for different people across different contexts. Utilizing a greater variety of research methods to capture people’s perspectives can provide a deeper comprehension of the construct, including different dimensions of wellbeing (Lomas et al., 2020; Alexandrova and Fabian, 2022).

Yoga is an embodied practice looking at the individual through different dimensions of existence, including physical, emotional, mental, social and spiritual, in a holistic way (Vivekananda, 2012; Ramirez-Duran et al., 2022). Yoga can also entail the evolution of every dimension of the individual where practitioners can express themselves to their optimal potential, in relation to themselves, to others and higher planes (Vivekananda, 2012). Since yoga involves the development of multiple dimensions of human functioning through a system of practice, regular yoga practitioners’ perceptions can offer a finer and more integrated view of wellbeing. Within the array of modern yoga styles, Ashtanga Yoga is a system encompassing a methodical practice underpinned by philosophical elements, thus providing both consistency and the potential to illuminate conceptualizations of different dimensions of wellbeing from an alternate embodied worldview.

Aligned with an ideographic approach and based on constructivism and phenomenology, this article expands on previous research on regular Ashtanga Yoga practitioners’ conceptualizations of wellbeing (Ramirez-Duran et al., 2022) by exploring in greater detail and depth five dimensions of wellbeing (i.e., physical, emotional, psychological, social, spiritual) within the same sample. Here, the main aims were (1) to examine each of these dimensions as perceived by practitioners, (2) to understand the relationship between these dimensions, and (3) to explore how these perceptions contribute to the comprehension of each dimension and wellbeing as a whole. To articulate the latter, we synthesized findings into a preliminary model. While our findings are mostly informed by wellbeing and yoga literature, we also draw upon relevant literature across a range of other disciplines.

### Wellbeing revisited

At an individual and subjective level, wellbeing has been broadly defined as feeling good and functioning well (Deci and Ryan, 2008; Keyes and Annas, 2009; Huppert, 2014; Adler and Seligman, 2016), and has been widely acknowledged as a multidimensional construct (Friedman and Kern, 2014; Manning and Fleming, 2019; Chia et al., 2020). However, several issues have been raised around the conceptualization of wellbeing. The vast array of definitions, dimensions, measurement tools, and overlapping terms across disciplines pose a significant challenge to researchers, scholars, and practitioners (Linton et al., 2016; Simons and Baldwin, 2021). Thus, deciding what wellbeing means and how it should be measured in a particular context can become a complex task with far reaching implications (Linton et al., 2016; Alexandrova, 2017; Simons and Baldwin, 2021; Alexandrova and Fabian, 2022).

There is an increasing awareness within science and philosophy that the conceptualization and evaluation of wellbeing can drastically vary depending on the context and the criteria used in each case (Hone et al., 2014; Khaw and Kern, 2014; Alexandrova, 2017). Since the construct of wellbeing simultaneously describes and evaluates what it is, its value is bounded to who is describing and making that evaluation (Alexandrova and Fabian, 2022). On the one hand, researchers and scholars can adopt particular and often unacknowledged perspectives, priorities and preferences, imbuing their own set values in their approach to wellbeing (Kern et al., 2020). On the other hand, research participants and stakeholders usually assess their own wellbeing using self-reported measures based on elements that not necessarily represent what they value the most (e.g., Khaw and Kern, 2014; Hone et al., 2015; Black and Kern, 2020). Narrowing the measurement of the construct to elements that may be irrelevant or impertinent for people’s circumstances can hinder our comprehension of the nuances of wellbeing.

As developing a sound empirical foundation for wellbeing depends on clarifying how it is defined and the type of evaluations that are undertaken, it is essential to tackle this challenge by acknowledging its evaluative content and involving all participants in the description and evaluation process (Alexandrova and Fabian, 2022). This translates into exploring participants’ perceptions about what wellbeing means to them, what are the elements that compose their definition and the criteria to assess its fulfilment. Scholars have recently highlighted the value of diversity emerging from a wide spectrum of conceptualizations and the richness provided by different research methods and interdisciplinarity in wellbeing science (Kern et al., 2020; Lomas et al., 2020; Mitchell and Alexandrova, 2021). Current research is shifting towards this direction. Wellbeing conceptualization has been studied from the perspective of young people (Bharara et al., 2019) and workers in New Zealand (Hone et al., 2015) using prototype analysis, Aboriginal communities using yarning circles (Garvey et al., 2021), Chinese international students in Australia using a phenomenographic approach (Huang et al., 2020), and regular yoga practitioners’ across different countries using reflexive thematic analysis (Ramirez-Duran et al., 2022). These approaches not only provide a richer and deeper understanding of what wellbeing means, but also confirm what scholars and researchers have proposed: that individual subjective wellbeing is a multidimensional construct.

### Dimensions of wellbeing

Before examining these multiple facets, it is worth clarifying the meaning of the term “dimension” and what it entails. Dimensions have been frequently defined within the literature in terms of variables or “conceptual subscales” included in self-reported measurements, such as self-acceptance, meaning or positive affect (Linton et al., 2016). While some scholars have referred to the term “dimension of wellbeing” as specific elements or variables that compose wellbeing (e.g., Ryff and Keyes, 1995; Linton et al., 2016), others consider it as broader aspect or area of life (e.g., Keyes and Waterman, 2003; Manning and Fleming, 2019; Chia et al., 2020). The first view is illustrated through wellbeing list theories (Alexandrova, 2017), which include a selection of features that create wellbeing, such as the PERMA framework (Seligman, 2011). The second view is represented by facets of individual functioning, such as emotional wellbeing (Diener et al., 1999, 2009), psychological wellbeing (Ryff, 1989, 2014), social wellbeing (Keyes, 1998), or spiritual wellbeing (Fisher, 2011). For purposes of this study, we adopt the latter approach and define the multidimensional nature of wellbeing first in terms of these facets of individual functioning. However, we still acknowledge that each of these facets or dimensions are comprised of specific elements, and thus, are also multidimensional on their own right. Hence, we define the term dimension of wellbeing as a set of variables that can be grouped under one area or domain of individual subjective wellbeing, which may be interrelated to each other to different extents.

When looking at the variety of features across self-report tools and the emerging body of research on lay conceptualizations of wellbeing, the multidimensional nature of wellbeing is evident, including physical, psychological, emotional, social, cultural, economic and spiritual domains (Linton et al., 2016; White et al., 2016; Butler et al., 2019; Manning and Fleming, 2019; Chia et al., 2020; Huang et al., 2020). This is consistent with recent research on regular yoga practitioners’ conceptualizations of wellbeing, unearthing the relevance of physical, emotional, psychological, social, spiritual, and ethical wellbeing as distinct dimensions according to participants’ views (Ramirez-Duran et al., 2022). This is further aligned with a range of wellbeing models focusing on one or more of these dimensions, such as the Complete Mental Health model (Keyes, 2005) including emotional, psychological and social wellbeing, and the Maori model of health *Te Whare Tapa Whā* (Durie, 1999) encompassing physical, mental, social, and spiritual health. The term social–emotional wellbeing has also been used to describe Aboriginal and Torres Strait islanders’ wellbeing as comprising physical, spiritual, mental, emotional and social domains, with mental and emotional composing one domain (Dudgeon et al., 2017). Noteworthily, labelling wellbeing as emotional, psychological, or mental represent different ways of setting boundaries to conceptualize subjective wellbeing (Ryff et al., 2021). Emotional wellbeing is usually considered as the presence of positive affect and the absence of negative affect (Diener et al., 1999, 2009), while psychological wellbeing is often considered as functioning well (Ryff et al., 2021). Yet, we acknowledge that these distinctions are social constructions and do not imply that dimensions are siloed.

Here, we have chosen five dimensions that focus on subjective individual functioning and that are well documented in the wellbeing literature and in previous research with yoga practitioners. We highlight not only the need to examine wellbeing through a multidimensional lens, but also to further explore dimensions through people’s eyes by using a mixed-methods approach. Since yoga is considered a method that binds body, mind, and spirit in ways that can evolve every dimension of an individual (Vivekananda, 2012), exploring regular yoga practitioners’ perceptions of wellbeing may provide novel insights and a more detailed understanding about the dimensions of wellbeing.

### Yoga, wellbeing, and dimensions of human existence

Yoga is an integrated system of theory and practice estimated to have been originated in the Hindu Valley sometime between 2 and 5,000 years ago (DeMichelis, 2004; Iyengar, 2005; Foxen and Kuberry, 2021). Often conceptualized as mainly a physical practice in the Western world (Singleton, 2010), yoga was originally conceived as a method that can use the body to still the mind and access a higher state of consciousness, where individual and universal consciousness become integrated (Iyengar, 2005; Larson, 2012). Thus, in yoga traditions the physical, mental and spiritual dimensions of the individual are intricately interconnected. According to the Vedic yoga text *Taittirīya Upaniṣad*, human existence encompasses five sheaths known as the *pancha-kosha* model (Vivekananda, 2012; Feuerstein, 2013). These layers are nested into each other ranging from denser (i.e., physical body) to more subtle (i.e., bliss body) levels of being, representing interrelated dimensions of the individual (Feuerstein, 2013). Given that yoga is a system of personal inquiry and experience, a regular practice can facilitate the realization and evolution of these layers of existence, leading to the awareness of humans’ spiritual nature, and influencing the relationship with others and the world (Vivekananda, 2012). Therefore, considering the experiences of regular yoga practitioners can be key to understand the benefits of yoga in relation various dimensions of individual wellbeing, including physical, mental, social and spiritual.

Although its betterments are well recognized in its original environment, it was only a century ago that yoga reached Westernized practitioners and researchers (Singleton, 2010; Ivtzan and Jegatheeswaran, 2015). Since the year 2000, research on yoga has exponentially grown, with over 4,000 publications documented by 2018 ranging across a variety of disciplines (Gupta et al., 2018; Gururaja et al., 2020; Poornima and Surulinathi, 2020). With a substantial research output to date, an important number of systematic reviews and meta-analyses have grouped studies investigating the influence of yoga on a range of health and wellbeing outcomes (e.g., Cramer et al., 2013, 2015; Field, 2017). However, the empirical literature suggests that yoga has been mainly used and studied as a short-term intervention to improve disease rather than to enhance wellbeing (e.g., Cramer et al., 2014; Gururaja et al., 2020). Furthermore, only specific dimensions of wellbeing have been addressed, with only a handful of studies investigating multiple dimensions in regular practitioners (e.g., Riley and Park, 2015). Therefore, focusing on yoga as a practice instead of as an intervention in a sample of established practitioners may provide interesting views on the different layers of optimal human functioning.

Ashtanga Yoga (AY) is one specific type of yoga derived from the *Hatha Yoga*, a tradition commonly understood as postural yoga for body and mind purification and vitality (Liberman, 2008; Jois, 2018). In short, AY can be defined as movement meditation (Maehle, 2006). AY is also defined as a method involving three distinct features known as *trishtana*, meaning “three places”: the practice of physical postures (i.e., *asana*) while directing attention to a specific gazing point (i.e., *drishti*) and using free breathing with sound (Jois, 2016). Another relevant element is *vinyasa*, which refers to the synchronization of *asana* and breath, as well as threading the *asanas* throughout the practice. AY is also known as *Ashtanga Vinyasa Yoga* to distinguish it from *Patanjali’s Ashtanga Yoga.* The latter is a philosophical framework underpinning the practice of yoga which comprises eight (i.e., *ashta*) limbs (i.e., *anga*). Although different, it has been argued that these eight elements can be learnt alongside, mirrored and experienced through the yoga practice (Maehle, 2006; Jois, 2016). For purposes of this research, we will use the term AY to refer to the body–mind practice that can be underpinned by Patanjali’s philosophical framework.

The AY practice consists of a sequence of postures usually taught progressively and using a one-on-one approach by a teacher in a group setting (i.e., Mysore style class), allowing the practitioner to slowly develop their own practice while building steadiness and strength (Jois, 2018). The AY method includes a set sequence of postures, starting with sun salutations (i.e., *surya namaskar A and* B) and standing postures before starting primary series. Practitioners can move gradually from primary to intermediate to advanced series. The practice typically includes Mysore style classes and Sanskrit-counted led classes four to 6 days a week, often including chanting of opening and closing mantras, resting days, and the practice of other elements such as energy seals (i.e., *bandhas*) and breathing techniques (i.e., *pranayama*) (Jois, 2016; Jois, 2018). The systematic nature of the AY practice can provide a higher consistency and uniformity across practitioners compared to other styles of yoga, thus addressing some methodological issues raised in previous yoga research (Riley and Park, 2015).

### The current study

The current study follows up on previous research exploring regular AY practitioners’ (AYPs) perceptions of wellbeing in general (Ramirez-Duran et al., 2022), by diving into their conceptualizations of five different dimensions of wellbeing (i.e., physical, emotional, psychological, social, spiritual). The present study focused firstly on exploring regular AYPs’ conceptualizations about these five dimensions of wellbeing, secondly on how these dimensions are interconnected, and lastly, on how these perceptions can contribute to understanding relevant qualities of each dimension and overall wellbeing. The dimensions included in this study were selected *a priori* both based on the aforementioned research and on existing wellbeing and yoga frameworks and research, that point to the relevance of physical, emotional, psychological, social, and spiritual domains to individual subjective wellbeing. Although defined *a priori*, a set definition of each dimension was not provided to participants. Following an ideographic approach and based on constructivist and phenomenological perspectives, our aim was to understand how each dimension is conceptualized by participants, based on their own experiences and perceptions. We used a predominately qualitative mixed-methods design, integrating quantitative and qualitative elements during data collection and analysis.

## Materials and methods

### Participants

This study utilized a purposeful sample from a broader research project focusing on AY and wellbeing, which involved 352 participants fluent in English and/or Spanish across 42 countries. Data were collected between April and August 2020 using an online survey (see Supplementary Table 1). Here, we included 137 participants who considered themselves regular AYPs and provided their conceptualizations for the dimensions of wellbeing. While the criterion of “regular practice” was deemed by these practitioners themselves (see Supplementary Table 1), we further considered frequency (i.e., days per week), expertise (i.e., years of practice and asana sequence), and consistency of the practice (i.e., rated in a scale from 1 to 10). From this sample, 131 participants provided their definitions for the five dimensions of wellbeing, four participants described four dimensions and two participants defined three dimensions. Respondents were recruited online through social media platforms (i.e., Facebook, Instagram, Reddit, LinkedIn) and electronic communication from yoga studios and/or teachers. Participants included a diversity of demographic and AY characteristics. All procedures were approved by the University of Melbourne’s Human Research Ethics Committee (protocol #1955377.1).

Most participants identified as female (80.59%), Caucasian (56.47%), between 25 and 44 years old (80.59%), living in an urban area (62.94%), and holding a tertiary education or postgraduate degree (85.88%). The majority of respondents had been practicing regularly for one to 10 years (67.16%) between 5 and 6 days a week (54.74%) for 1 to 2 h per session (79.57%), and in a consistent manner (86.13%). Most practitioners followed a Mysore style (91.25%) and practiced either primary (48.18%) or intermediate (44.53%) series, typically including *vinyasa* (97.08%), *drishti* (94.89%), *bandhas* (91.97%), and free breathing with sound (89.78%). Most participants considered yoga philosophy relevant (87.60%) and engaged with it by reading books and texts (83.21%), following social media (54.01%), reading blogs (53.28%), and watching videos (51.82%) at least once per week (59.86%). Most practitioners reported the practice or experience of the eight elements from the AY framework to a high extent (i.e., seven or above in a 10-point Likert scale). *Asana* was the element practiced to a higher extent by most participants (94.89%), followed by the practice of *yama* (89.05%), *pranayama* (84.67%) and *dharana* (78.83%). The vast majority of practitioners perceived that their AY practice positively contributed to their wellbeing, especially to their physical (93.43%), emotional (92.70%), and psychological (88.32%) wellbeing, reporting high levels of wellbeing (i.e., scored seven or above in a 10-point Likert scale) in these areas accordingly (see Supplementary Tables 2–7 for details).

### Measures

An online survey comprising quantitative and qualitative items was designed to measure aspects of yoga practice, wellbeing, health, personal traits, and demographic information (see Supplementary Table 7), with the survey offered in both English and Spanish. The present study incorporated questions about participants’ AY practice, demographic information, and six open-ended questions exploring their perceptions of five dimensions of wellbeing (i.e., physical, emotional, psychological, social, spiritual) and other dimensions that they estimated relevant and were not included in the survey (see Supplementary Table 1).

### Data analyses

Participants logged their responses using Qualtrics survey software,^1^ which were then exported to NVivo (version 12) software for analysis. We analysed responses to the six open-ended questions in two stages. The first stage entailed overviewing participants’ perceptions by examining word frequency, creating word clouds, word trees, and summary tables (see Supplementary Tables 8–13) to capture conceptualizations in their own words for each dimension of wellbeing. While concise, this type of quantitative analysis provides a general understanding of data before diving into a deeper qualitative analysis (McNaught and Lam, 2010; Eichstaedt et al., 2021). This analysis was part of the first step of the next stage, and enabled researchers to familiarize with the data while providing readers with a general impression to better understand the reflexive and interpretative process.

**Figure 1:**
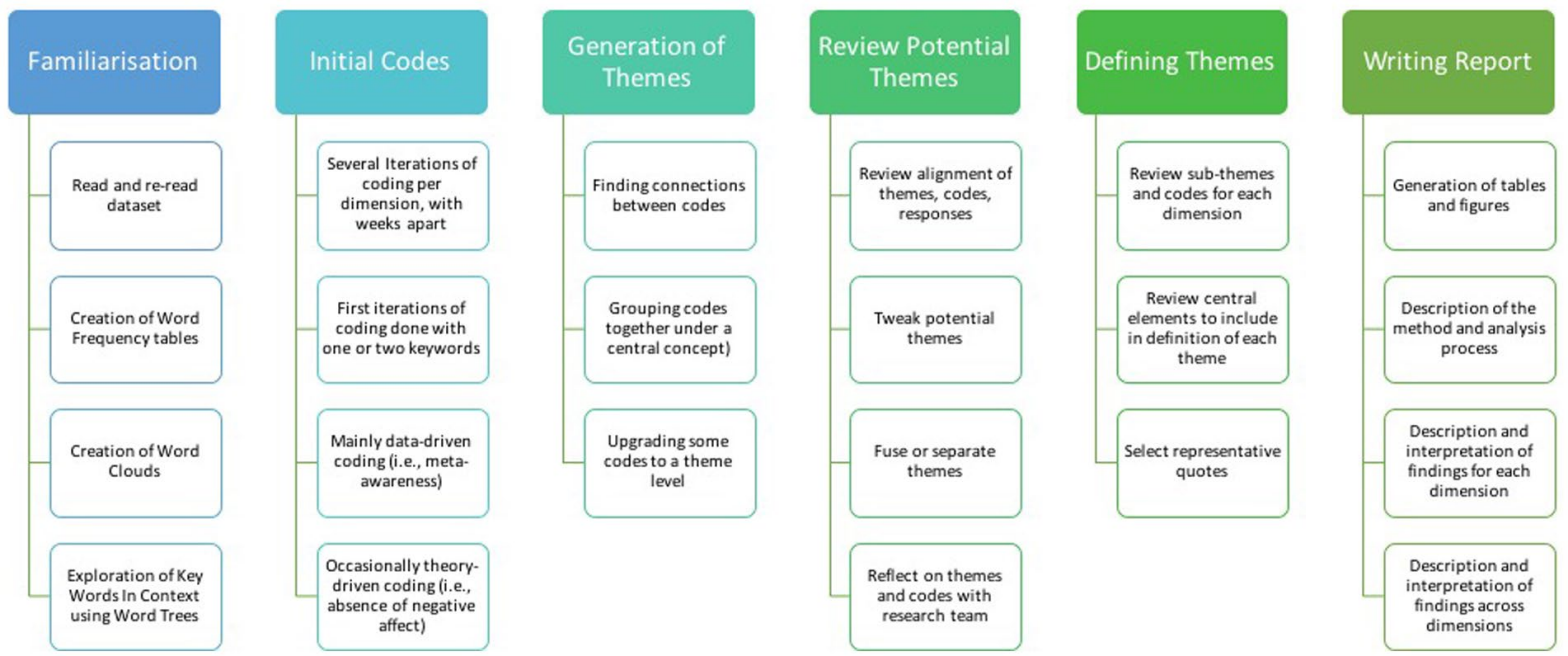
Application of the six steps of RTA in this study.

The second stage used Reflexive Thematic Analysis (RTA; Braun and Clarke, 2012, 2021) to identify, reflect on, and interpret meaningful patterns across participants in a systematic manner. Following Braun and Clarke (2012, 2021) guidelines, we applied six recursive steps to analyze data, including (1) familiarization with the data, (2) generation of codes, (3) construction of candidate themes, (4) review of candidate themes, (5) definition and naming final themes, and (6) writing a report. Details about these steps are provided in the next section (see Figure 1). This six-fold process was repeated for each dimension of wellbeing, aiming to generate key themes for each one of them. We then mapped themes for each dimension to visualize and identify shared patterns of meaning across the five dimensions.

### Generation of codes and definition of themes

Consistent with a constructivist epistemology, meaningfulness of information across participants was regarded essential to create codes and themes. In RTA, the role of the researcher is central to illuminate and shape the data analysis, with beliefs, values, background, and interests actively informing the analysis (Terry and Hayfield, 2020; Braun and Clarke, 2021). Aligned with RTA, the analysis was completed by the lead author because of her expertise across psychology, wellbeing, yoga, and AY in particular. Reflexivity was performed throughout the analysis process by the lead author on her own, but also in collaboration with the two other authors to sense-check ideas and explore alternate interpretations of the data (Braun and Clarke, 2021; Byrne, 2021). While the creation of the survey and the interpretation of findings were influenced by our backgrounds, it also allowed us to utilize a common language with AYPs and to identify key elements provided by the respondents.

**Figure 2:**
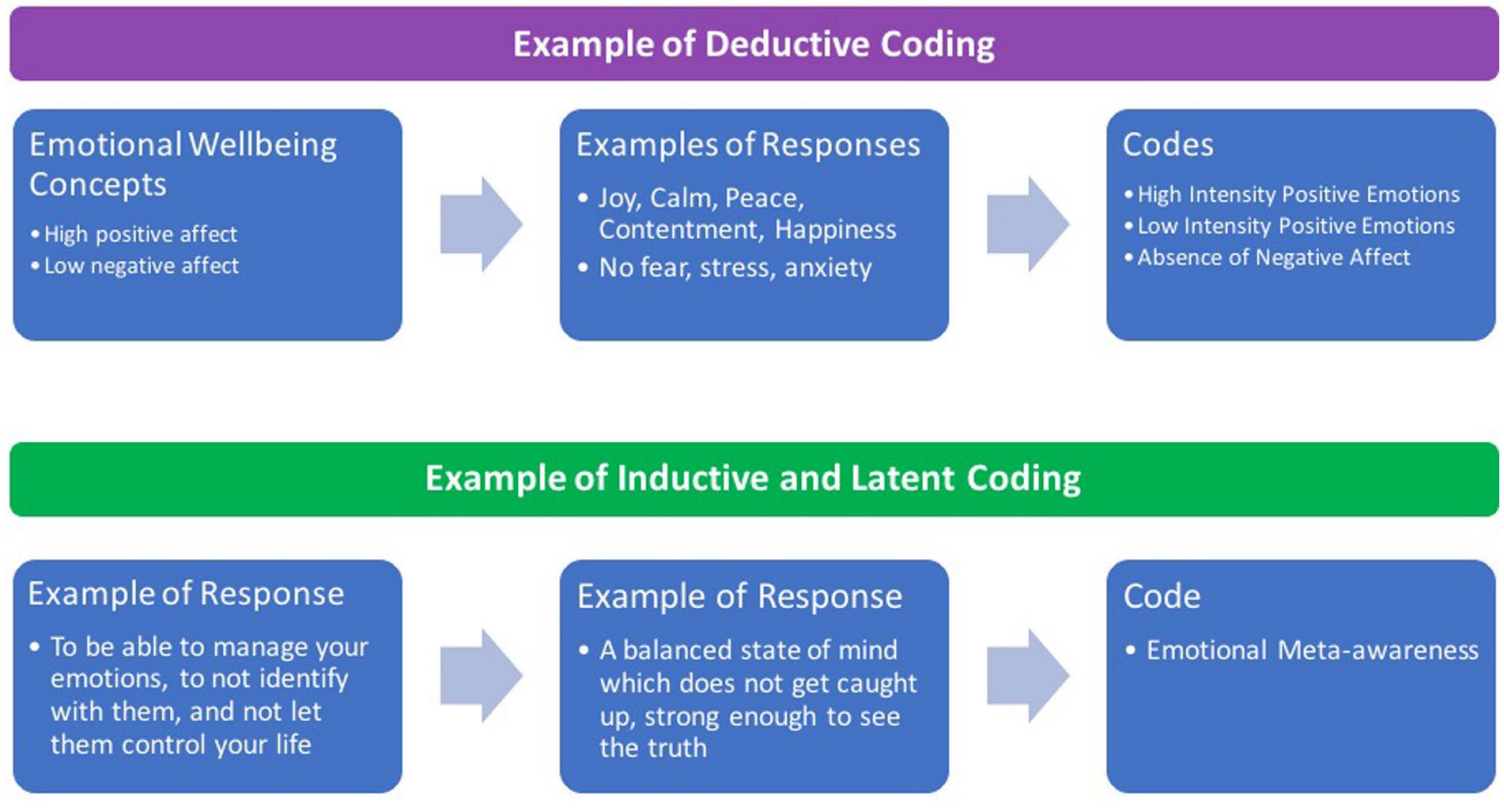
Examples of deductive and inductive coding process.

After familiarizing with the data, initial codes were generated using both inductive and deductive approaches, as well as blending latent with semantic coding. These blends are well recognized and used within RTA (Braun and Clarke, 2021; Byrne, 2021). Inductive coding (i.e., data-driven) was the predominant approach used in this study, to best capture the perspectives of participants. Deductive coding (i.e., theoretically-driven) was applied by considering a range of wellbeing theories and yoga philosophical frameworks, yet codes were not created prior to data analysis, consistent with the reflexive approach (Braun and Clarke, 2021). For example, within the emotional dimension, codes such as “Emotional Meta-awareness” and “Emotional Steadiness” represented an inductive process, whereas the codes “Absence of Negative Affect” and “High Intensity Positive Emotions” represented a deductive approach (see Figure 2). Latent coding was widely predominant in relation to semantic coding, by actively interpreting relevant shared underlying meanings across participants’ responses. A semantic approach was followed when meaningful semantic information was found across participants. For instance, while the code “Agency and Autonomy over the Physical Self” in the physical dimension primarily involved interpretation of responses across participants, the code “Acceptance of Pleasant and Unpleasant Emotions” within the emotional dimension was considered at a descriptive and explicit level.

**Figure 3:**
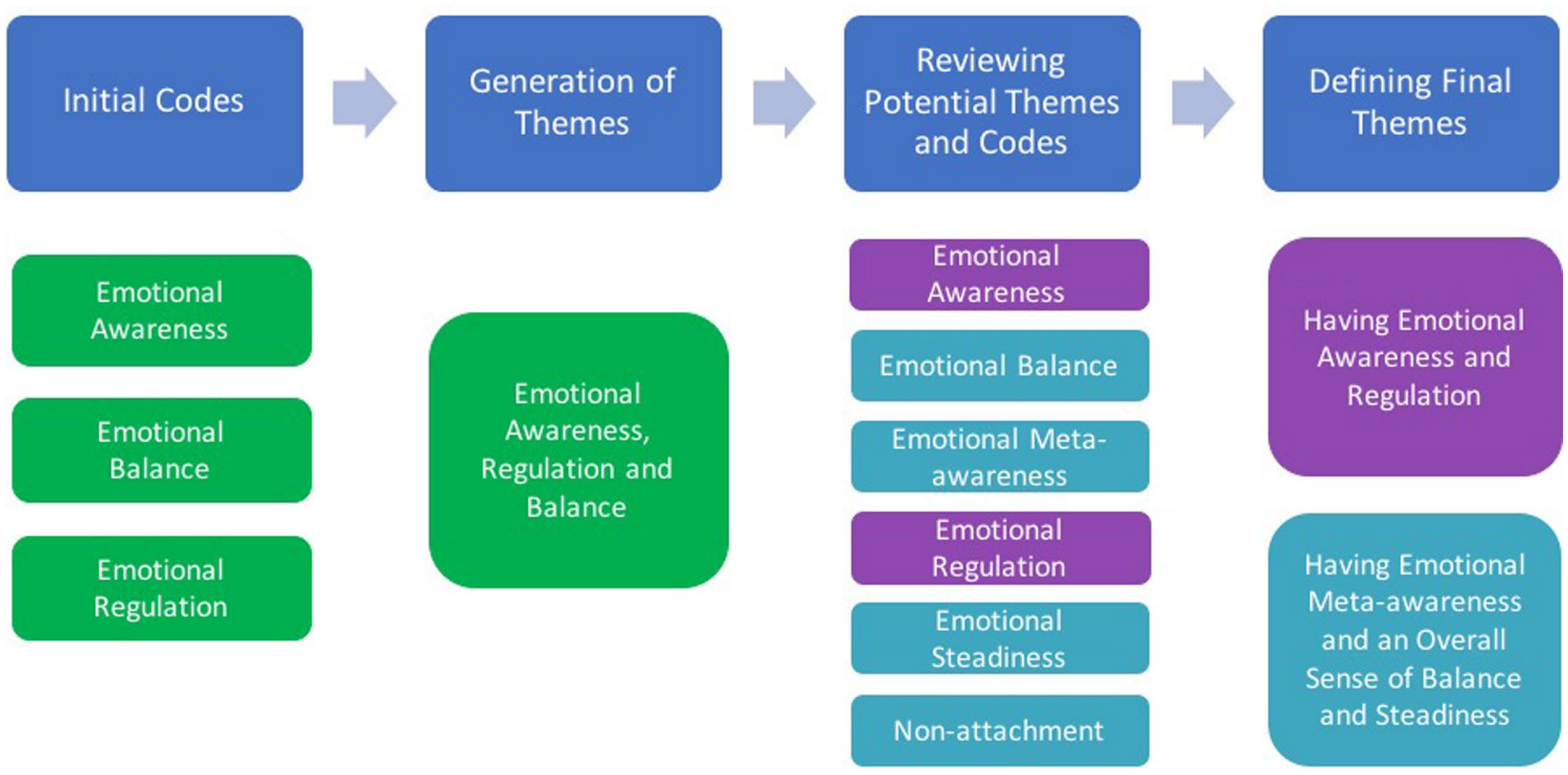
Example of the process to generate, review, and define themes.

After generating codes, candidate themes were constructed and then reviewed before defining the final themes included in this manuscript. An example of this process is provided in Figure 3 for two themes from the Emotional Wellbeing dimension. Initially, we created codes to represent the concepts of emotional awareness, emotional regulation, and emotional balance in participants’ responses. A preliminary theme was created to reflect the process of identifying, managing, and balancing emotions as part of emotional wellbeing. After several iterations of coding and reviewing themes, we refined codes to better reflect participants’ perspectives. For example, the existing code “Emotional Awareness” was defined as “being aware of feeling, of what emotions are being felt, and understand emotions” and the new code “Emotional Meta-awareness” was created to represent perceptions about observing emotional states, processes, and responses. Emotional Awareness and Emotional Regulation were regarded as one theme concerned with knowledge and abilities about emotional states, whereas other themes reflected a more transcendental and holistic perspective about emotions.

## Results

### An overview of the linguistic landscape of wellbeing dimensions

Before delving into the reflexive analysis of themes for each dimension, we first offer a descriptive analysis of the words used for each dimension of wellbeing. Exploring words and their frequency was incorporated as part of the familiarization with the data stage of RTA. Typically, familiarization encompasses reading several times the whole dataset until the researcher becomes closely related to the data (Byrne, 2021). We argue that exploring word frequencies and key words in context can provide an overview of the dataset when there is a considerable number of responses and responses are not very extensive. We also believe that this approach may provide the reader with a general view of findings and ease them into each theme and their discussion.

**Figure 4:**
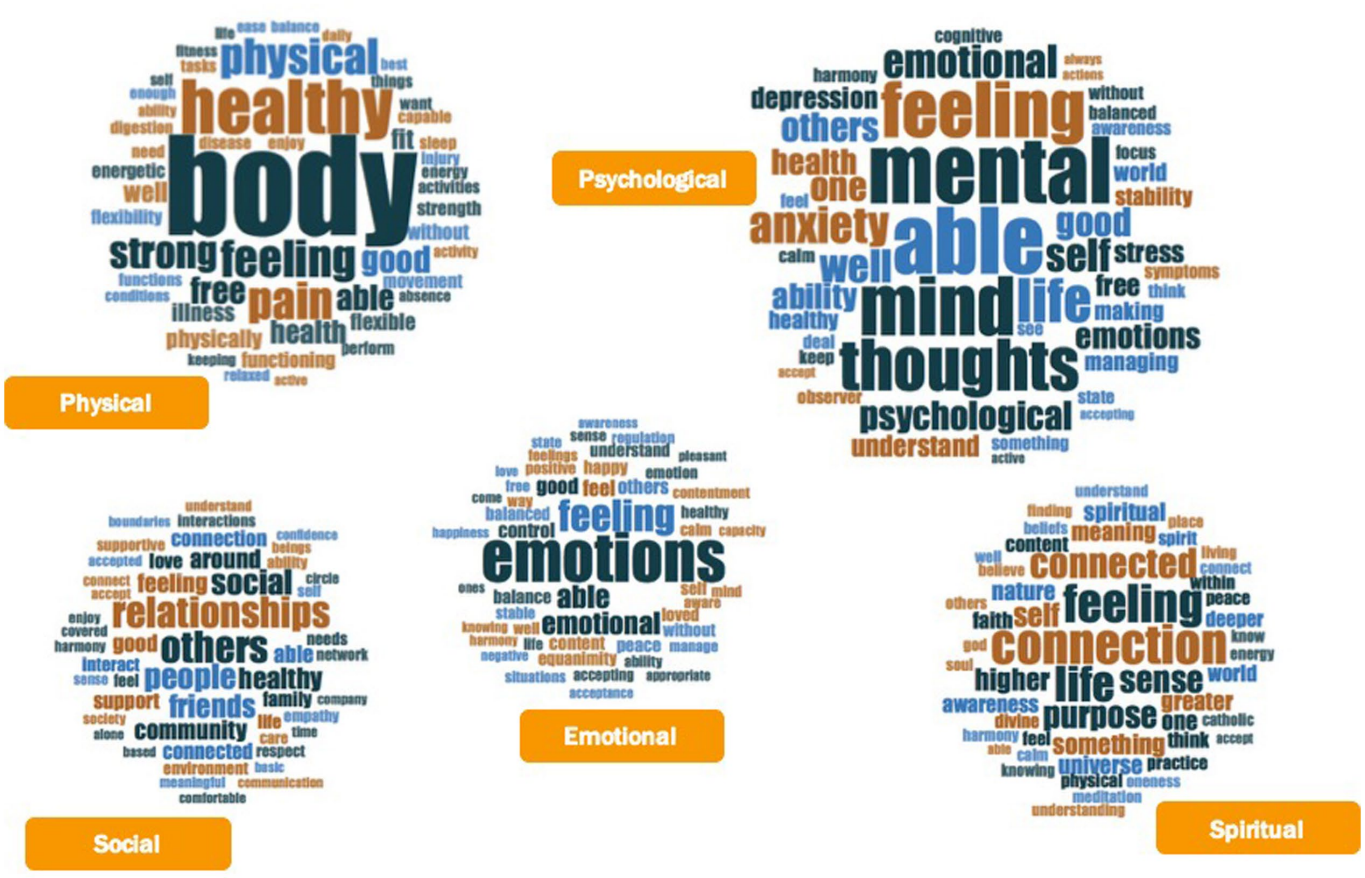
Word clouds for physical, emotional, psychological, social, and spiritual dimensions of wellbeing as defined by regular AYPs.

Responses varied in length (min = 1, max = 60) and participants used an average of eight to nine words to define each dimension of wellbeing (see Table 1). The 50 most frequently used words by regular AYPs to define each dimension of wellbeing are depicted in Figure 4. Larger words represent a higher frequency (min = 3, max = 73), without a specific meaning for color. The frequency and distribution of words across dimensions varied (see Supplementary Tables 8–12 for details), thus producing some word clouds with larger font size (e.g., physical and psychological dimensions) and others with smaller size (e.g., emotional, social and spiritual dimensions).

**Table 1:** Overview of regular AYPs’ responses word counts for each dimension of wellbeing.

	Physical	Emotional	Psychological	Social	Spiritual
N	137	136	136	135	133
Min.	1	1	1	1	1
Max.	32	46	39	30	60
Mean	8.4	8.4	8.9	8.3	9.3
Median	7	6	6	6	7

We identified constellations of common words within and across dimensions. For example, words such as “good,” “healthy,” and “well” were commonly used across physical, emotional, psychological and social wellbeing. The use of words referring to impaired health, such as “illness,” “pain,” and “injury” were frequently mentioned across physical and psychological wellbeing. Participants also used words such as “able” and “ability,” as well as “feel” or “feeling” across the five dimensions to conceptualize wellbeing (see Supplementary Tables 8–12 for details on each dimension). In general, the term “able” referred to either experiencing something or performing an action, while “feeling” mainly referred to an experience (see word trees in Supplementary Table 13).

### Shared meanings across regular AYPs within wellbeing dimensions

Applied to each dimension of wellbeing separately, RTA entailed actively reflecting on and interpreting codes across responses by developing themes beyond semantic representations. The reflexive analysis yielded four themes for each wellbeing dimension (see Table 2). This section aims to succinctly discuss themes within each wellbeing dimension, highlighting distinctive elements related to yoga, and provides an overall definition for each dimension from regular AYPs’ perspectives. For writing purposes, we intentionally organized themes by building upon previously described themes while maintaining their internal consistency and distinct narrative, to create a coherent narrative of the data for each dimension of wellbeing in this report, as suggested by RTA guidelines (Braun and Clarke, 2012; Byrne, 2021). Extending on this analysis, we examined overarching key elements across wellbeing dimensions by identifying common patterns in themes from different dimensions, which will be addressed in more detail in the discussion section.

**Table 2:** Definitions of wellbeing dimensions and their themes, with quotes from regular AYPs and frequencies.

**WB Dimensions**	**Themes**	**Representative quotes**	***N***	% (*)
Physical	Being free from and able to manage physical suffering	“Freedom from illness and pain”	27	15.61%
		“Free of disease or illness or at least able to manage any illness or symptoms in a constructive manner”		
		“Managing any medical conditions and staying healthy”		
	Having a functioning body, responsive to internal and external demands	“Able to perform daily tasks fully”	25	14.45%
		“To have a body that is capable of doing the things you need to do to stay connected and in balance”		
		“Well functioning body systems”		
	Having a sense of health and positive physicality	“Feeling fit and strong in my body”	81	46.82%
		“A feeling of wellness within the physical body (i.e., fit, strong)”		
		“Healthy, active, strong, flexible”		
	Consciously and actively inhabiting the body	“Looking to make the best choices and decisions to promote my best physical being”	40	23.12%
		“Being able to do the things I want to do with this body”		
		“Being content in my own skin”		
Emotional	Being able to experience positive affect and the absence of negative affect	“Feeling calm and content”	57	30.81%
		“Feeling safe and at peace”		
		“Having a positive outlook, not feeling down or other negative states of being. Feeling buoyant”		
	Being able to embrace emotions, valuing negative affect and challenges	“An understanding and acceptance of emotions and the importance of both pleasant and unpleasant emotions”	24	12.97%
		“Not necessarily feeling joyful all the time, but being able to appreciate that all emotions, positive and negative, play a role in making life what it is”		
		“Internally balanced and able to cope with external situations”		
	Having emotional awareness and regulation	“Able to feel, understand and process emotions and ability to regulate them when necessary”	51	27.57%
		“Being in touch/aware of your emotions and displaying them in appropriate manners”		
		“Being able to understand the emotional state you are in, and cope with changes to that in a good way”		
	Having emotional meta-awareness and an overall sense of balance and steadiness	“The state of ones emotions - is there stability? Are they positive/negative? How quickly do they return to homeostasis?”	53	28.65%
		“A balanced state of mind which does not get caught up, strong enough to see the truth”		
		“Being at peace with my emotions, live them and let them go”		
Psychological	Being free from and being able to manage mental suffering	“Absence of psychological conditions such as depression and anxiety”	30	15.31%
		“Well in the psyche. Active participation with healing and processing”		
		“Not experiencing the symptoms of mental illness, or being able to monitor and be aware of it.”		
	Having a strong sense of identity and self	“Not identifying myself with darkness in my mind, being able to accept the shadows”	62	31.63%
		“Being comfortable with who you are and what you are doing”		
		“Having resilience to deal with the many curveballs that come your way. Never doubting that you are strong enough to deal with them”		
	Experiencing a sense of mental steadiness	“The mind can go towards anxiety and for psychological wellbeing, I believe that is essential to learn how to breath and calm the mind”	58	29.59%
		“Having an even temperament when faced with all of life’s offerings”		
		“Being strong and balanced mentally and in my thoughts”			
	Engaging in the contemplation of the self	“How you interpret reality, how you respond to the external world, how you imagine yourself to be in the world”	46	23.47%
		“Working to become the observer. To truly understand where my psychological baseline is and then be the observer of my “psychology” outside of that”		
		“Being able to take the observer’s viewpoint when it comes to thoughts, emotions, bodily sensations, events and experiences.”		
Social	Presence of social connections and social competence	“Having people around me”	38	17.76%
		“Friends, family, significant others”		
		“Being able to interact appropriately in a social environment”		
	Feeling connected and attuned to others and to the environment	“Being connected and of service to my community”	42	19.63%
		“Connected with others in meaningful relationships”		
		“Functional and positive interpersonal relationships, connection to a community, contribute to others’ welfare, receive help and support”		
	Having high quality interpersonal relationships	“Our capacity to foster meaningful relationships and sense of belonging within a chosen community/society”	99	46.26%
		“Having a strong group of friends that you trust and can rely on, and in return providing the same for them”		
		“The ability to form healthy mutually enriching relationships, altruism, empathy, kindness”		
	Engaging in the contemplation of the social self and relationships	“Actively co-creating with others, using community to mirror back to one’s self areas for improvement”	35	16.36%
		“Balancing social life and essential alone time”		
		“Having healthy boundaries”		
Spiritual	A sense of connection to oneself and to an individual purpose and meaning in life	“Knowing your purpose and living (towards) it”	81	38.03%
		“Feeling your own energy, find calm amidst the ego”		
		“Being able to live a life of grounded ease, comfortable in your own skin. Being accepting of your strengths and weaknesses, but also improving/fine tuning actions as well. This is a constant work in progress, daily honest reflection is required.”		
	A sense of connection to others and to the environment	“How I’m connected to the world outside of my mind/body”	28	13.15%
		“Feeling connected to the Earth and all its beings”		
		“Feeling like you belong to a bigger whole than just yourself (nature for me)”		
	Engaging in the contemplation of the self and connection to the transcendental self	“Keeping a strong relationship with my soul, contemplate myself and life itself”	52	24.41%
		“Connection with the higher self”		
		“Connected to what is, not identifying with the ego”		
	A sense of connection to the divine and to a higher purpose and meaning	“Feeling the presence of the Supreme around you”	52	24.41%
		“Our connection with, and awareness of, what exists beyond our individual and collective physical existence”		
		“Able to practice and apply dharma teachings in daily life”		

*Percentages were calculated based on the total mentions (*N*) within each dimension.

#### Physical dimension of wellbeing

The first theme was “*being free from and able to manage physical suffering”* and was defined as experiencing the absence of pain, illness, and injuries, and having the ability and capacity to deal with and/or recover from them when they are present. For example, one participant noted physical wellbeing was being “free of disease or illness, or at least able to manage any illness or symptoms in a constructive manner.” This theme may represent a baseline where wellbeing as a positive attribute can emerge from.

The second theme was *“having a functioning body, responsive to internal and external demands.”* This was defined as the capacity to perform basic physiological functions, with bodily organs working properly and a body able to recover and to complete tasks and leisure activities someone needs and wants to do. In words of one participant, physical wellbeing is about “your body and how well it functions in its everyday tasks like activity, fighting infection, digestion, excretion, and so on.” Another participant spoke about being “able to perform daily tasks fully.” This theme extends on the previous one, looking at physical wellbeing as a functioning body rather than only the absence of ailments.

The third theme within this dimension was “*having a sense of health and positive physicality,”* which was defined as experiencing musculoskeletal abilities and features including strength, endurance, flexibility, energy levels, confidence, and balance. One participant illustrated this as “feeling capable and healthy and free in my body,” while another portrayed it as being “healthy, strong, and vital.” This theme encapsulates positive physical attributes as experienced by practitioners, and the elements we identified mirror features of the AY practice.

The fourth theme we identified was *“consciously and actively inhabiting the body,”* defined as having a sense of embodied ownership characterized by a feeling of ease, contentment, autonomy, and agency. This embodied feeling was also characterized as being facilitated by bodily awareness and meta-awareness, and by having the ability to make choices for healthy living. For instance, one participant mentioned “having full command of all body functions,” while others wrote about having a “healthy body with a healthy attachment to what the body presents” and “eating well, exercising, getting good sleep.” This theme depicts physical wellbeing both as emerging from an embodied experience of physical awareness and as a contemplative attitude for deciding the best choices for the physical body.

From the eyes of regular AYPs, we suggest that physical wellbeing can be defined as a multilayered experience ranging from the absence of physical ailment, the ability to manage and raise above suffering, and the ability to experience positive physicality, while engaging in physical awareness and contemplation to make the best choices for healthy living. Some elements within this dimension can be paralleled to those described in the wellbeing literature, such as autonomy, agency, and competence (Ryan and Deci, 2000) and in the wellness literature, such as strength, illness prevention, physical activity and nutrition (Hettler et al., 1980). Other elements seem to emerge from the embodied experience of yoga, such as movement, flexibility and physical activity, contentment, and a sense of ease in the body, and awareness and contemplation of the physical self.

#### Emotional dimension of wellbeing

The first theme in this dimension was “*being able to experience positive affect and the absence of negative affect.”* This can be defined as the ability to feel a range of emotions and affects, primarily pleasant with low intensity (i.e., calm, peace, contentment) but also high intensity (i.e., joy, happiness). For instance, one practitioner portrayed emotional wellbeing as “contentment, lack of anxiety, and a sense of compassion” while another spoke about “calmness, happiness, and motivation.” This theme represents emotional wellbeing mainly as the presence of pleasant emotions, which is consistent with existing literature.

The second theme was *“being able to embrace emotions, valuing negative affect and challenges,”* which was defined as having the ability to feel acceptance towards unpleasant affective states and identifying opportunities for growth. For instance, one participant described emotional wellbeing as “an understanding and acceptance of emotions and the importance of both pleasant and unpleasant emotions.” Another practitioner defined it as being “able to be with whatever emotions arise and stay at an even keel.” The ability to accept and positively appraise negative affect and states adds another layer to the conceptualization of emotional wellbeing, similar to what was proposed in the second wave of positive psychology (Wong, 2011).

The third theme within this dimension was “*having emotional awareness and regulation,”* defined more specifically as having the ability to identify, feel, understand, accept, process, manage and respond to a variety of emotions. For example, one participant spoke about emotional wellbeing as the “awareness of emotions and able to express them freely (to oneself and others).” Another practitioner perceived it as “being steady, handling feelings/reactions maturely, communicating openly with others.” This theme represents the conscious process by which emotions exist as a lived experience, including sensations, thoughts, and behaviors. Although there is a robust body of literature on emotional literacy and emotion regulation (e.g., Gratz and Roemer, 2004), this has not been explicitly incorporated into subjective wellbeing models.

The fourth theme we identified was *“having emotional meta-awareness and an overall sense of balance and steadiness.”* This was defined as being able to stay emotionally stable and balanced throughout time and to return to balance from oscillating emotional states, by observing ebbs and flows of emotional states and by not identifying with those emotional states. One practitioner conceptualized emotional wellbeing as being “able to manage your emotions, to not identify with them, and not let them control your life,” while another described it as meaning “that emotions emerge without generating unconscious or automatic reaction.” This theme adds another layer of complexity to the emotional wellbeing dimension by revealing the relevance of a contemplative attitude in the conceptualization of wellbeing.

According to regular AYPs’, we suggest that emotional wellbeing can be understood as embracing all emotions, being able to experience pleasant affect and positively value unpleasant affect by cultivating emotional awareness, regulation, and meta-awareness. Within this dimension, positive affect is concordant with extensive literature stating that this is an essential part of subjective wellbeing (e.g., Desbordes et al., 2018). Yet, elements such as low intensity positive emotions, acceptance and equanimity, emotional awareness, and meta-awareness may reflect aspects that emanate from yogic practices and frameworks.

#### Psychological dimension of wellbeing

Similar to the physical dimension, the first theme was “*being free from and being able to manage mental suffering.”* We defined this theme as experiencing the absence of mental illness and having the tools and skills to deal with those conditions and life challenges when they emerge. Participants often mentioned freedom from depression, anxiety, and stress and although managing them was relevant, specific strategies were not specified. One participant spoke about psychological wellbeing as being “free from suffering mental illness,” while another defined it as “mentally healthy enough to cope with the challenges of daily life and to have strategies or treatments to help when needed.” This theme represents a ground-level for wellbeing in this dimension, from which other attributes can take place.

The second theme was *“having an integrated sense of identity and self**,”*** which we defined as experiencing a clear representation of who one is as well as a coherent and stable perception of that individuality, including traits, skills, and behaviors. Here, we identified three key subthemes: (1) *“knowing and accepting oneself,”* (2) *“regulating oneself,”* and (3) *“having a sense of control and ownership of oneself.”* For instance, one practitioner conceptualized wellbeing as “self-acceptance and self-love,” another as “being able to contain myself in moments of crisis and being able to reflect on myself,” and another participant as “strength and confidence.” This theme speaks about the relevance to cultivate positive attributes to develop a healthy ego and is aligned with wellbeing models including elements such as autonomy, resilience, and self-acceptance.

The third theme within this dimension was *“experiencing a sense of mental steadiness,”* which we defined as being able to direct and focus attention and experience equanimity, balance, and a sense of contentment. For instance, one practitioner described psychological wellbeing as “feeling good and also accepting the bad mood, remaining neutral,” while another defined it as having “a clear mind, able to easily focus and be silent whenever I need to.” This theme can be viewed as a bridge between the previous and the next theme, where developing a steady mind may play a role in the sense of self and the ability to contemplate the self.

The final theme in this dimension was *“engaging in the contemplation of the self.”* We defined this as consciously practicing meta-awareness and meta-cognition on aspects of the self by becoming the observer of states, thoughts, and emotions, and by understanding the psychology of the self and choosing how to behave. For example, while one practitioner wrote about “understanding what triggers you, what is unhelpful to your mental health, and knowing what tools aid your wellbeing,” another described psychological wellbeing as “being able to see yourself from a distance.” Within this theme, practices such as breathing, yoga, and meditation were mentioned as means to achieve wellbeing. For instance, one participant portrayed wellbeing as the “consciousness of states of mind and the ability to smooth them” and another illustrated through “contemplative practices and community.” Similar to previous dimensions, a contemplative attitude appears to be a key element in wellbeing.

Following regular AYPs’ perceptions, we suggest that psychological wellbeing can be defined as the dynamic interrelation between the absence of mental ailments and the experience of a strong and integrated sense of self. Here, the self is able to direct and still the mind while engaging in contemplation and managing mental suffering and challenges. While elements concerning the freedom from mental illness and its management (Seligman et al., 2004) and the sense of self are well established in the psychology literature (e.g., James, 1890; Kohut, 1971, 1977), other elements may specifically reflect yoga practices. For example, mental concentration and steadiness, are often referred to as developing a “one-pointed mind” in the AY arena, facilitated by drishti and breath. Other elements such as equanimity, inner-work or self-transformation, and contemplation altogether may specifically mirror contemplative aspects of yogic traditions.

#### Social dimension of wellbeing

The first theme we identified in this dimension was the *“presence of social connections and social competence*.” This theme suggests that, at a fundamental level, social wellbeing is about having social connections and experiencing a sense of social competence by having adequate interpersonal skills. For example, one participant spoke about “the ability to form healthy mutually enriching relationships,” while another wrote “having a good social circle, having people to talk to.” This theme may represent the first layer of social wellbeing, where basic elements need to be in place to provide a fertile ground to grow.

The second theme was *“feeling connected and attuned to others and to the environment,”* which we defined as experiencing a sense of pleasant connection to the external world, including other beings and the environment, through a sense of community, contribution, and harmony. For example, one participant conceptualized social wellbeing as “feeling connected to friends/family/communities” and another as being “in harmony with the environment and others.” This theme highlights the feelings that are experienced by an individual when in interaction with their external world.

The third theme was *“having high quality interpersonal relationships.”* We conceptualized this as the experience of meaningful, healthy, and strong interpersonal relationships characterized by a range of reciprocal features such as trust, honesty, acceptance, kindness, love, and compassion. These relationships also featured the ability of engaging in supportive social relationships at a micro (i.e., interpersonal) and macro level (i.e., community and society), finding a healthy balance between giving and taking. One participant defined this dimension as “having a healthy group of people around you to hear and support you, share and connect with.” Another practitioner spoke about “functional and positive interpersonal relationships, connection to a community, contribute to others’ welfare, receive help and support.” This theme encapsulates the essential qualities of positive relationships through the perspectives of regular AYPs.

The last theme was *“engaging in the contemplation of the social self and relationships,”* which we defined as the ability to observe the quality of interactions and the self in social contexts, cultivating an attitude of acceptance towards others and oneself. This included the ability to draw boundaries and balance social life with alone time. This can be illustrated in statements such as “openness in the way to accept every creature,” “enjoying the company of others while being content with myself” and “using social interactions for knowing ourselves and to control our egos.” This theme emphasizes an attitude of observance and suggests a healthy attachment with others while using social interactions as an opportunity for self-knowledge and growth.

From the regular AYPs’ perspectives, we suggest that social wellbeing can be defined as both the objective presence of having social connections and the subjective experience of feeling connected and competent with others and the environment, through stable, nurturing mutual interactions, while developing the capacity of contemplating those interactions and oneself in the social realm. While the relevance of social connections, high quality connections and social support is well documented in the positive psychology literature and included in many wellbeing models (e.g., Ryff, 1989; Keyes, 2007), other features included in this dimension might provide an alternate view. For instance, concepts such as harmony and balance, positively valuing solitude, experiencing contentment and engaging in contemplation may be related to Eastern frameworks and contemplative practices such as yoga.

#### Spiritual dimension of wellbeing

The first theme we identified in this dimension was “*a sense of connection to oneself and to an individual purpose and meaning in life.”* We defined this as consciously engaging in self-knowledge, self-improvement, and self-growth, exploring personal life purpose, and cultivating positive mental and emotional states, including contentment and equanimity. One practitioner defined spiritual wellbeing as “knowing myself and being in line with my purpose.” Another participant explained it as “feeling at peace within myself.” This theme may reflect that the basis of building spiritual wellbeing, resides within and involves inner work.

The second theme was “*a sense of connection to others and to the environment,”* which we defined as feeling connected to other beings, seeing other beings as oneself, and feeling as part of or as one with nature. For instance, while one participant spoke about “feeling like you belong to a bigger whole than just yourself (nature for me),” another practitioner mentioned “a state of connection, unification with you, with the earth, with the smiling beings on the planet, and with the galaxy, the universe, and multiverses.” This theme captures the belief that spiritual wellbeing is about the lived experience of interrelatedness and oneness with the external world.

The third theme was “*engaging in the contemplation of the self and connection to the transcendental self.”* This was defined as developing a meta-awareness and meta-cognition of the self by contemplating the self and human existence, not identifying with the ego, and connecting with a higher self, or a higher state of consciousness. Some statements from participants that define this dimension of wellbeing are “keeping a strong relationship with my soul, contemplate myself and life itself,” “feeling connected to my higher self” and “listening to our spirit through meditation and a life that matches our energy.” This theme may represent a layer bridging the psychological self and the connection to divinity, where a greater or higher awareness emerges by contemplating existence.

The final theme in this dimension was “*a sense of connection to the divine and to a higher purpose and meaning.”* We conceptualized this as experiencing a feeling of connection to a higher realm and believing in a higher purpose, which may guide the personal path. Practicing yoga, meditation, chanting, and ethical principles emerged as tools to cultivate spiritual wellbeing. One practitioner defined spiritual wellbeing as “a connection with Oneness – which for me is God,” while another participant spoke about “being aware of a universal energy around us.” Another practitioner wrote about “meditation, chanting and study of yogic scriptures.” This theme highlights an awareness of and the interrelation of human existence with a divine source.

From AYPs’ perspectives, we suggest that spiritual wellbeing can be defined as a lived experience of awareness, connection, meaning, and purpose across different realms, including the individual self, other beings, the environment, and a divine source, along with practices and beliefs that cultivate such connection. The themes identified within this dimension mirror existing conceptualizations of spiritual wellbeing, especially in terms of connection with a divine realm, meaning and purpose (e.g., Fisher, 2011). Specific elements seem closely related to yogic frameworks and may bring further insights into these conceptualizations. For instance, the cultivation of awareness and contemplation through yoga-related practices and the distinction of the self and a higher self, appear to be central for spiritual wellbeing.

#### Other wellbeing dimensions

Participants also had the opportunity to share their perceptions about other areas of wellbeing that were relevant for them. We identified three main topics across participant responses (n = 15). *Intellectual and creative wellbeing* was included by three participants. This dimension was defined in terms of cultivating learning, challenging ways of thinking and fostering creativity. For example, one practitioner wrote about being “engaged in activities in which one can learn and grow, challenge thinking and evolve.” *Environmental wellbeing* was proposed by four participants and it was defined as taking individual and collective action to respect and protect ecosystems, and live in harmony within them. For instance, one participant spoke about “living in harmony with other sentient beings and with your surroundings.” *Moral and ethical wellbeing* was included by five practitioners and was conceptualized as considering and living by principles and beliefs for the common good. This dimension was intertwined with environmental wellbeing and consideration for following a plant-based diet. While one practitioner referred to this as “living your life thinking about what is right for everyone, above personal interests,” another spoke about “veganism is important to me and yoga, as I see them going hand in hand.” Finally, one participant mentioned *energetic wellbeing* as “cultivating the energy body” and two others mentioned *sexual wellbeing* and *personal freedom and autonomy*, but without providing any definition.

These additional dimensions enrich the perspectives provided for the other five dimensions and may suggest further connections to yoga frameworks. For example, intellectual wellbeing and energetic wellbeing may reflect two layers of human existence as proposed by yoga traditions, namely the wisdom body and the energy body that are part of the *pancha kosha* model (Feuerstein, 2013). Additionally, *environmental wellbeing* and *moral wellbeing* may be interrelated to aspects of psychological, social and spiritual wellbeing, since it considers ethical principles and behaviors, connection to a larger context, and the integration of the external and the internal world. Furthermore, it can be argued that they also reflect elements contained in yoga traditions. For example, moral wellbeing can be connected to the first two elements of *Patanjali’s Eightfold Path*, namely *yamas* and *niyamas*. These represent ethics in relation to relationships with others and individual moral codes for self-improvement (Vivekananda, 2012).

## Discussion

Findings from our RTA extend on previous research on lay people’s conceptualizations of wellbeing in general, by undertaking an in-depth exploration of five dimensions of wellbeing that have been previously documented in the positive psychology literature. By using alternate research methods (i.e., ideographic, mixed-methods) and drawing upon further literature on wellbeing, psychology, philosophy, yoga traditions, and contemplative practices, as well as using a systems lens to discuss individual functioning (Kern et al., 2020), this article offers a deeper and broader perspective on these five dimensions in particular and of wellbeing in general. This section not only provides further insights about each dimension on its own, but it also extends our understanding of wellbeing by looking at regular AYPs’ shared meanings across dimensions.

### Shared meanings within and across wellbeing dimensions

**Figure 5:**
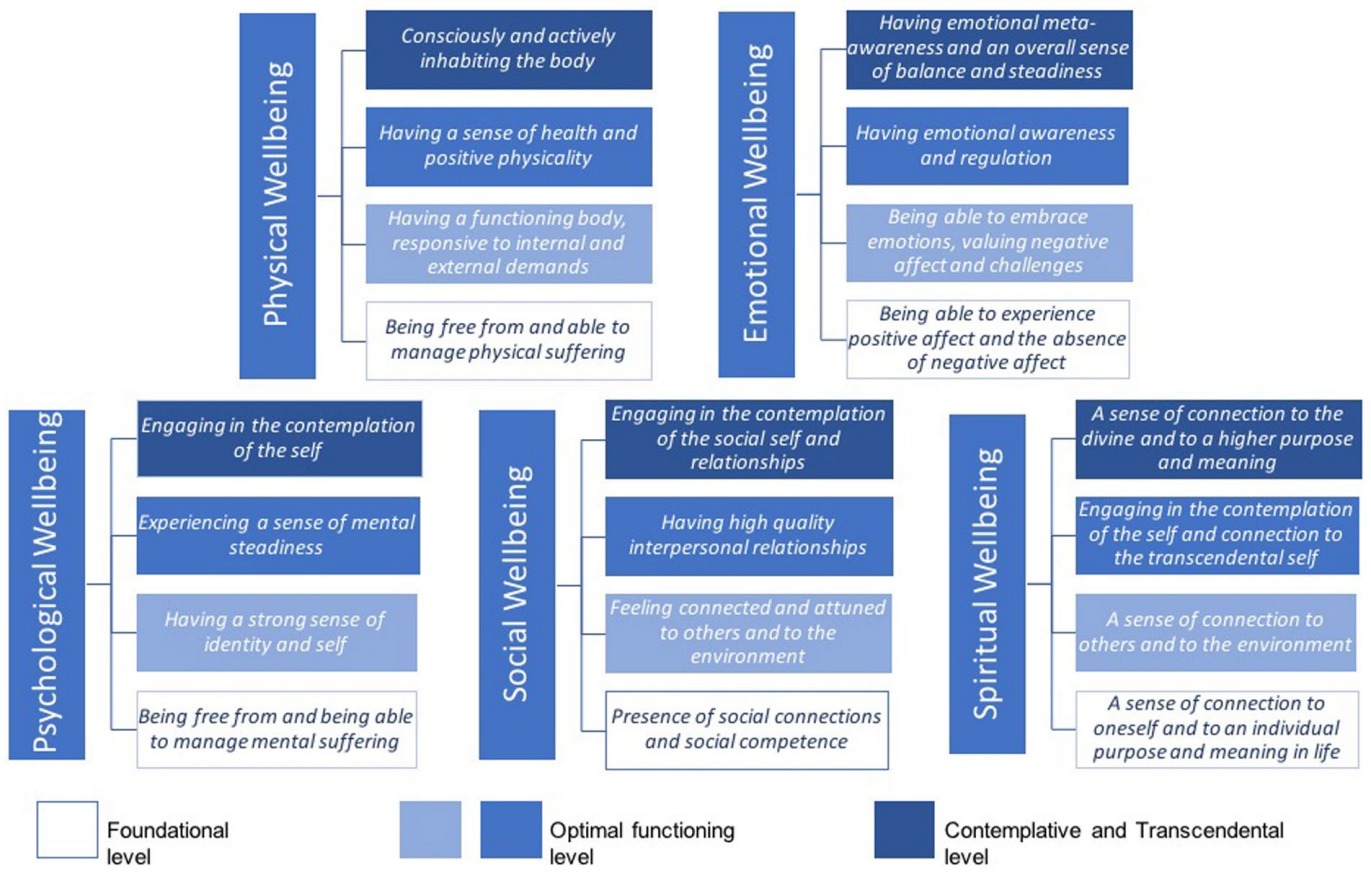
Levels of wellbeing dimensions.

**Figure 6:**
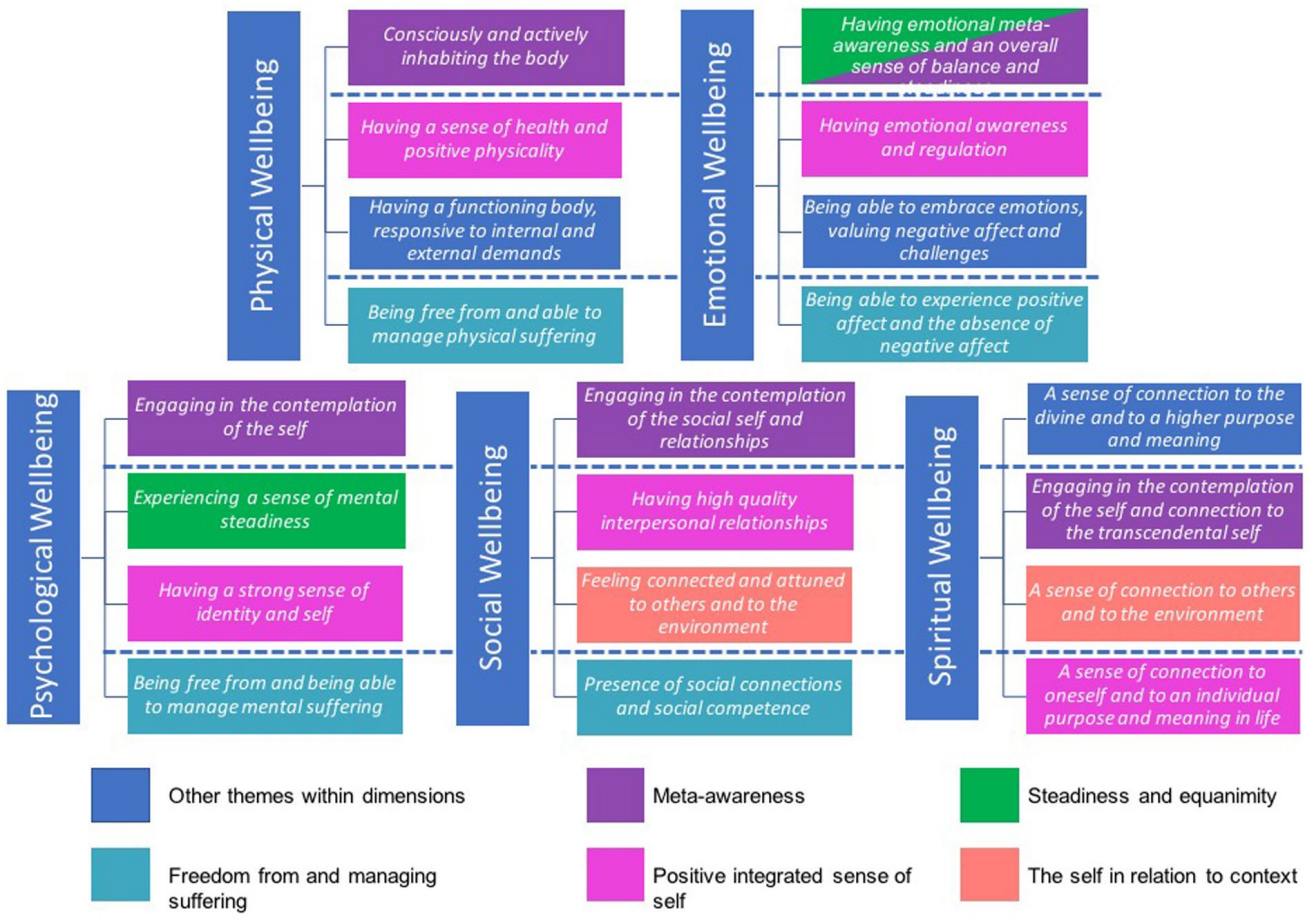
Common themes across dimensions of wellbeing.

Based on the previous analysis, we identified two main trends in relation to dimensions of wellbeing. One is that each wellbeing dimension can be understood as multileveled, starting from a level comprising basic, ground-level elements (e.g., lack of symptoms) to more complex levels involving different types of functioning and engagement of the self with the internal and external world (e.g., personal growth, transcendence). The other trend is that wellbeing dimensions share common themes, expressed in particular forms depending on the dimension of individual functioning that is being observed, but still reflecting the same core idea. The former trend can be visually represented by using a vertical plane within each dimension to locate each level of functioning, with the foundational level at the base of each dimension (see Figure 5). The latter can be visualized from a horizontal plane by looking at common themes across dimensions that share the same core concept and are represented by using the same color (see Figure 6).

Noteworthily, while other dimensions proposed by a smaller group of participants were highly regarded, we pursued the integration of shared meanings across dimensions within the larger set of participants by mainly considering the five dimensions of wellbeing (i.e., physical, emotional, psychological, social, spiritual). However, our analysis still found links and overlaps between those dimensions proposed by participants and the themes found within the five dimensions of wellbeing. For example, environmental wellbeing was mentioned by participants as part of social and spiritual dimensions (theme 2), moral wellbeing was mentioned as part of spiritual dimension (theme 4), and energetic wellbeing was mentioned as part of physical dimension (theme 3). Sexual, intellectual and creative wellbeing were not identified as themes in any of the five dimensions, and thus may represent aspects of individual functioning that could be further explored on their own in future research. The next sections will discuss key aspects from these trends, by exploring each level and discussing the common themes that we found across dimensions within each level. We then continue to integrate this into an emergent model by looking at these trends in perspective.

### The levels of functioning in wellbeing dimensions

From our thematic analysis, we found that each dimension of wellbeing included aspects that could be organized by levels or layers of individual functioning. Figure 5 summarizes the themes for each dimension of wellbeing while illustrating three layers: a foundational level, an optimal functioning level, and a contemplative and transcendental level. While we have used three levels to broadly group common themes that represent each layer of wellbeing, we acknowledge that there may be other ways to organize and interpret this information. Similarly, we acknowledge that there may be other possible ways of organizing, interpreting and integrating the themes found across the five dimensions of wellbeing. Our analysis and integration of information does not pursue reaching an exact or literal match between the themes across dimensions, but rather seeks to create meaning of common perspectives about what wellbeing encompasses throughout these dimensions. Despite being other plausible ways of organizing our findings, we regard this approach as the best fit to synthesize and integrate complex information. For each level of individual functioning, we provide a definition and discuss common themes, along with our interpretation on how and why these themes span across multiple dimensions.

#### The foundational level of individual functioning

This level represents a baseline for individual functioning, where certain issues, barriers or challenges may need to be either resolved or well-managed, as well as having the ability to experience basic positive attributes, to create a fertile ground for wellbeing to grow. While each dimension has a foundational level, this may qualitatively vary when comparing dimensions. Overall, this layer can be seen as comprising two main features. One is freedom from unpleasant or undesirable objective (e.g., injury) and subjective (e.g., negative affect) states. The other is that being able to manage those negative states when they are present, as well as the presence of basic positive attributes to cope and function, are fundamental to wellbeing. To capture the overall meaning of the overarching theme within this level and reduce complexity, we will simply name it *freedom from and managing suffering*.

The absence of illness or unpleasant states was common across physical, emotional, and psychological wellbeing. While this is more aligned with a pathogenic view of health, the absence of negative states in these dimensions indicates that this still appears to be relevant for people to experience wellbeing to a full extent (Weich et al., 2011; Keyes, 2014). While some people may refer to the objective absence of negative states or manifestation of observable symptoms (i.e., “not getting sick often” or “no aches”), others may refer to their subjective experience as being undisturbed (i.e., “not feeling stressed”). This is connected to the other common theme within this layer, which is having tools to manage adversity and being able to experience basic positive attributes. This is aligned with research suggesting that individuals can experience wellbeing even when they experience ailment (Keyes, 2014). While having coping tools was common across physical and psychological dimensions, basic positive attributes were common across emotional, social and spiritual dimensions. This suggests that an internal physical, emotional and psychological balance might be needed (i.e., absence of negatives plus coping skills) in addition to the presence of positive emotions, human connections, and sense of self.

The recurrence of this theme across dimensions suggests that there is an interdependence and continuity between them. This might be explained by three key concepts that are shared by all humans: the experience of suffering, the need for safety, and the need for connection. The concept of human suffering is well acknowledged within Eastern philosophies (e.g., Loy, 2016) and in the compassion literature (e.g., Neff and Davidson, 2016) as an experience shared across humanity that emerges from the attachment to our senses, thoughts and interactions. Having a sense of safety also appears to be fundamental to human functioning. When a threat is perceived, the sense of safety is thwarted. Literature on trauma offers an integrated view of this concept, showing that psychological safety arises when there is a sense of embodied safety signaled by visceral activity associated to emotions (Van der Kolk, 2017). Furthermore, while the need for safety can be facilitated or thwarted by significant relationships, the presence of human connections still appears to be fundamental for different levels of individual functioning. For instance, the polyvagal theory proposes that humans are equipped with a social engagement system able to identify safety or threat in the environment to regulate emotional and social behavior and potentially play a role in the embodied feeling of wellbeing (Porges, 2009; Sullivan et al., 2018).

Thus, the conceptualization of wellbeing of regular practitioners as the absence of negative states, the ability to manage them and to experience a baseline of positive attributes may reflect basic human conditions and needs. Furthermore, the recurrent themes also imply a continuum throughout individual dimensions. Since yoga is an embodied and contemplative practice able to regulate the nervous system, it has the potential to provide both a sense of embodied safety and freedom from suffering by acknowledging yet not identifying with the negative states that may arise (Van der Kolk, 2017).

#### The optimal functioning level

This represents the level of functioning where the cultivation of positive attributes and states take place and flourishing happens. This level is different from the previous one in that the foundational level implies a deficit viewpoint and here there is a focus on what goes beyond a baseline functioning. In every dimension we identified themes and sub-themes (see Supplementary Table 14 for details) comprising elements well documented in the positive psychology literature on individual subjective wellbeing. These include vitality (Huppert and So, 2013), positive emotions (Diener et al., 2020), autonomy (Ryan and Deci, 2006), self-acceptance (Ryff and Keyes, 1995), self-regulation (Vohs and Baumeister, 2016), meaning and purpose (Steger, 2012), and positive relationships (Heaphy and Dutton, 2008). The acknowledgement of positive qualities as essential to wellbeing from participants’ perspectives supports the importance of including a range of attributes that compose and contribute to wellbeing. The range and variety of elements evidences the diversity in which wellbeing can be defined and experienced, the relevance that each individual gives to particular elements, and the combination of elements that they regard as essential to them. This is consistent with what scholars have proposed about the conceptualization and measurement of wellbeing (e.g., Alexandrova and Fabian, 2022). Furthermore, breaking down wellbeing into dimensions for practitioners to conceptualize them separately provided a richer and finer view of the elements and qualities comprising each facet, as well as commonalities across them. While these elements are not exhaustively addressed in this paper, here we focus on three common themes systematizing such elements across dimensions.

One theme that appeared across all five dimensions was a positive and integrated sense of self. This points to the relevance of having an integrated sense of identity grounded in the body and the mind, and in relation to the context and the spiritual world. This suggests that in terms of wellbeing, the traditional Western view of the self as a mainly psychological construct should consider other dimensions and the interaction between them. For instance, from a Western approach it has been proposed that the self can be considered as a multilevel system comprising components, interconnections and processes at a physical, psychological and social levels (Thagard, 2012). This is consistent with views of regular AYPs that perceptions of wellbeing involve the development of the self in relation to the physical, emotional, psychological, and social realms. However, practitioners views extends on this by considering the self in spiritual terms, as a deep connection with all elements of the self. The above is aligned with the *pancha kosha* model rooted in yoga traditions, that considers the self in terms of sheaths and in terms of its spiritual nature (Vivekananda, 2012). The recurrence of this theme across all dimensions reflects the relevance of the development of the self in a holistic way for optimal levels of functioning.

Another shared theme at this layer was experiencing steadiness or equanimity. This was found across three wellbeing dimensions. Within the emotional dimension, equanimity was found in the shape of experiencing low intensity emotions and as acceptance of all emotions. In the psychological dimension it was found as part of mental steadiness. And in the spiritual dimension, it emerged as part of the cultivation of positive states within the self (see Supplementary Table 14). This implies that equanimity is relevant for wellbeing, that it can be experienced as impacting different human dimensions and thus not only a purely psychological phenomenon. This is in line with Western scholars who have argued that equanimity is an emotional regulation strategy (Juneau et al., 2020; Wojnarowska et al., 2020), and with an Eastern perspective bounding this even-minded state to spiritual and contemplative philosophies and practices (Desbordes et al., 2015). Furthermore, recent research has shown that equanimity can be considered a wellbeing outcome (Desbordes et al., 2015) and that people with less barriers to experience equanimity are more likely to have higher levels of mental wellbeing, mindfulness, self-compassion, and better emotional regulation (Weber, 2020). Thus, the integration of literature on equanimity with views of regular AYPs suggests that equanimity can be experienced across and play a key role in emotional, psychological, and spiritual wellbeing.

The third common theme was the self in relation to others and to the world. This was observed mainly across the social and spiritual dimensions, but also to some extent in the psychological dimension. This took the common shape of the presence of relationships and feeling a sense of connection to other beings and to the environment. Both relatedness and belongingness have been widely studied across various disciplines. Relatedness has been found as a central component of human motivation and essential for individual wellbeing (Ryan and Deci, 2000). The motivation for belonging has been deemed unescapable and innate to humans, with positive, lasting and significant relationships being fundamental for human wellbeing (Baumeister and Leary, 1995; Leary and Cox, 2008). Moreover, the underlying meaning of terms such as “feeling connected” or “having a sense of connection” speaks to an embodied perception that can be related to yoga. This is aligned with previous research on perceptions of outcomes of the yoga practice in regular practitioners suggesting that this feeling of connectedness may flow from the intrapersonal development attributed to the practice (Kishida et al., 2018). Therefore, the commonality of this theme across dimensions may indicate not only the relevance of others for wellbeing in these areas, but also how connectedness can potentially be experienced qualitatively different and flow from one dimension to another.

#### The contemplative and transcendental level of human functioning

This represents a level of individual functioning where individuals are able to observe and take perspective about themselves and their internal and external functioning. This level is distinct from the previous ones, since there is a different type of engagement of the self-regarding human existence. This layer suggests the importance of contemplation upon elements that are contained in the previous layers across different dimensions, while adopting an embracing attitude towards these elements and developing higher states of consciousness.

Although there is a considerable body of literature about this level of functioning within areas such as transpersonal psychology (Maslow, 1969) and Eastern philosophies, this has been far less addressed in contemporary wellbeing models. Usually, individual subjective wellbeing frameworks are focused on the previous layer, where the psychological self or ego operates (e.g., Ryff, 1989; Seligman, 2011). This layer, however, functions beyond this view of the self. Here, the observer or the transcendental self comes in, functioning in a higher level of human consciousness. This was reflected as a common theme across the five dimensions of wellbeing in the shape of meta-awareness, or the ability to contemplate the body, emotions, thoughts, oneself, social relationships and elements beyond the self. Interestingly, the spiritual dimension also mirrored this on its own, with its different levels representing the contemplation of different dimensions of existence.

Meta-awareness may explain why practices involving mindfulness, including yoga, can promote wellbeing (Gard et al., 2014; Hussain, 2015). Research suggests that meta-awareness seems to play a key role in health and wellbeing in terms of identifying symptoms and preventing illness (e.g., Hargus et al., 2010), becoming aware of the experience of hedonic wellbeing (Schooler and Mauss, 2010), and experiencing a connection beyond human existence (Dambrun et al., 2019). Yoga can to promote self-regulation through meta-awareness of bodily and mental signals and processes, thus integrating information from different sources and facilitating wellbeing at different levels of functioning (Gard et al., 2014). Conceptualizations of wellbeing provided by regular practitioners are aligned with this evidence, reinforcing the notion that meta-awareness occurs at different levels of individual functioning and is relevant to wellbeing.

### The levels in perspective: Integrating Western and Eastern views on human functioning

Based on regular AYPs’ conceptualizations of the dimensions of wellbeing, we have identified three levels of individual functioning offering a novel perspective on wellbeing and the interrelation between dimensions through five common themes. These common themes found throughout dimensions representing shared meanings across practitioners, were interpreted and integrated using an interdisciplinary lens. In this section, we aim to discuss the levels of individual functioning in relation to the concept of the self and wellbeing integrating Western and Eastern theoretical frameworks.

The three levels identified across dimensions in this study are consistent with Abraham Maslow’s Theory Z (Maslow, 1971), which posits that there are three levels of human functioning. The first level is defined as security and includes the deficiency needs (D-needs) of safety, connection, and self-esteem. The second level is defined as growth, contains the being needs (B-needs) of exploration, love and purpose. The balance between these two levels would lead to self-realization or optimal human functioning. Finally, the third level is defined as transcendence, which goes beyond self-realization (Maslow, 1971; Kaufman, 2020). The theory proposes that needs are interrelated, able to be achieved simultaneously and work together towards a higher purpose (Kaufman, 2020). This is similar to the first two layers that we found in our analysis, with the foundational layer mirroring a deficit realm and the optimal functioning layer reflecting a personal growth realm. Since the foundational and optimal functioning layers were represented within each wellbeing dimension and shared meanings were identified across dimensions, our view can provide a broader understanding on individual functioning in terms of deficiency and growth needs within each dimension of wellbeing.

In addition to the interplay between these two layers, we also found a higher level of human functioning that was regarded relevant for wellbeing from practitioners’ perspectives. This evolution towards a higher level of functioning, is both consistent with what is proposed in Theory Z (Maslow, 1971; Kaufman, 2020) and in the *pancha kosha* model (Vivekananda, 2012). Maslow (1967) suggested that self-actualizers were more likely to transcend, since they would have fulfilled D-needs and lived most of the time in the B-realm, working on personal development. This is aligned with the yoga perspective on the evolution of the self that leads to higher states of consciousness to ultimately transcend the self (Vivekananda, 2012).

**Figure 7:**
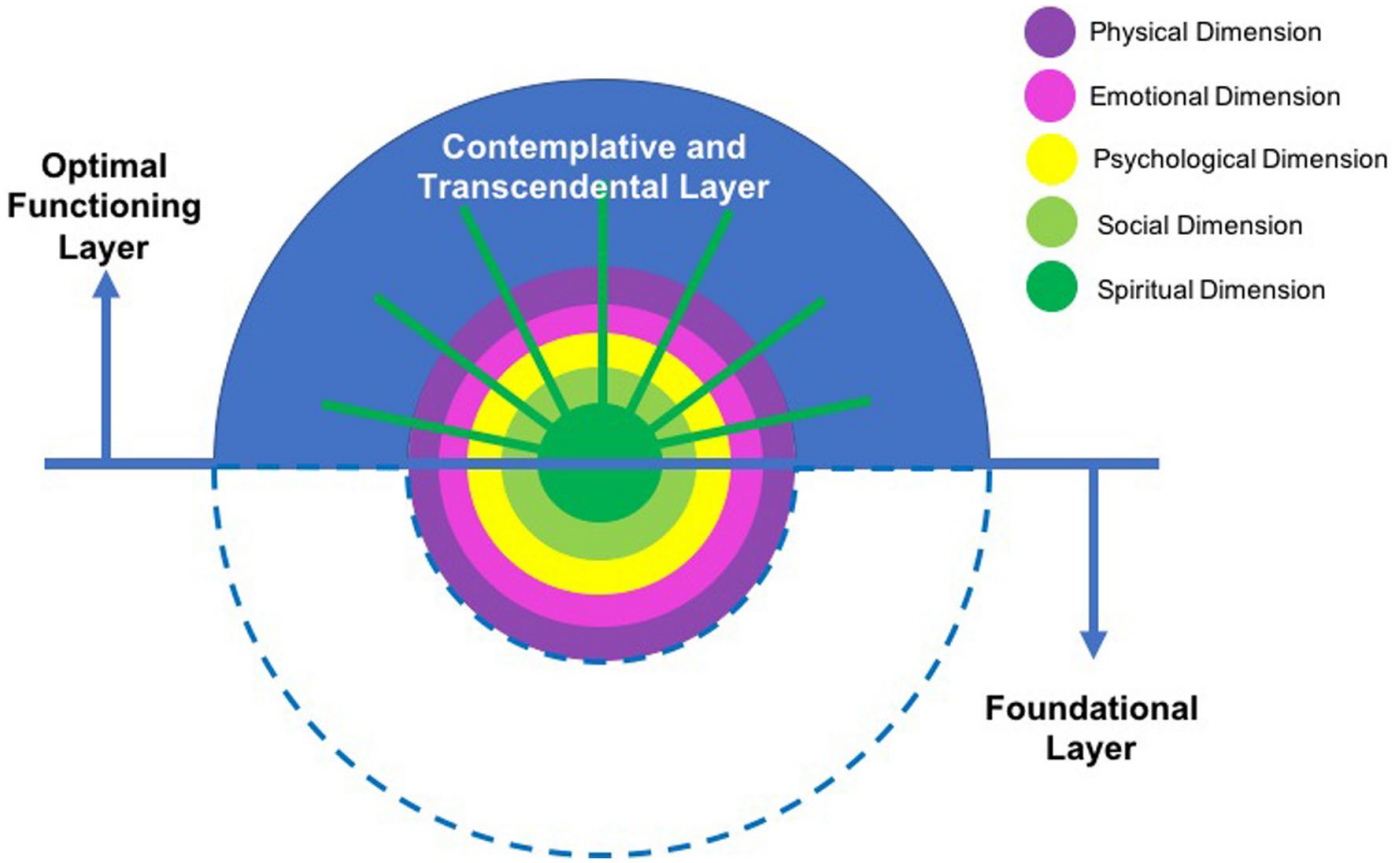
Levels of functioning in relation to dimensions of wellbeing.

Figure 7 synthesizes these planes of individual functioning along with the different dimensions of wellbeing that we explored in this study. The concentric circles represent the dimensions of individual subjective wellbeing. Dimensions have been nested within each other similar to what is proposed in the *pancha kosha* model with the five bodies and in systems literature (e.g., Bronfenbrenner and Ceci, 1994; Bronfenbrenner, 1995; Thagard, 2012). Here, we suggest this concentric representation reflects the interrelation and interdependence of dimensions rather than a hierarchal, linear or siloed perspective. The bottom half of the figure represents the foundational layer of functioning, while the top half represents the optimal layer of functioning. Each dimension can be represented at both these levels depending on whether wellbeing is being considered from a deficit or a growth point of view. This part of the graph represents the self. The arc represents the third layer, where the self is evolved or transcended. We have located the spiritual dimension at the core and extending towards the transcendental layer due to its presence in relation to all the other dimensions and its relevance to contemplation and transcendence. This view considers the self, and thus wellbeing, as an embodied and as a multifaceted construct.

### Implications and future directions

We explored five dimensions of wellbeing through the eyes of regular AYPs. While this sample predominately represented highly educated middle-aged women living in Westernized cultures, their views have provided a rich description of how each dimension of wellbeing can be defined and what elements may be central to their composition. Furthermore, shared meanings across dimensions provided a broader perspective on how these dimensions relate to each other and how they can be seen separately and holistically. This also allowed us to organize participants’ shared meanings in layers of individual functioning, represented by different activities and types of engagement of the self. This approach to wellbeing involves dynamism and complexity that might be better understood by looking at the self as a system in and of itself.

Although including elements from other dimensions such as emotional and relational, individual subjective wellbeing frameworks tend to use a psychological or mental approach to understand human functioning. While previous research with regular AYPs has highlighted the multidimensional, holistic, and embodied nature of wellbeing (Ramirez-Duran et al., 2022), the exploration of different dimensions revealed continuity of themes across dimensions. This reinforces the importance of embodiment and transcendence in wellbeing frameworks, with the implication of further exploring the self as an integrated system with different levels of functioning.

On the one hand, this might mean further investigating dimensions as nested cases from the physical to the spiritual realm, and what are the connections across. For example, we could define the self as a multilayered or multilevel system, comprised by different elements that exist on their own, in interaction with each other and with an external environment. Depending on which boundaries we use to define the self, we can look at different areas as a whole, and also in interaction with other areas of the self. Furthermore, considering elements such as meta-awareness and equanimity as central to wellbeing and to the evolution of the self may provide further insights about these connections.

On the other hand, this may imply exploring in more detail the levels of the self in terms of ways of knowing about the self and about wellbeing. We could define the self at the level of the experience and directly involving bodily systems such as sensorimotor, proprioceptive, and interoceptive systems. We could also define the self at the level of reflection and mental processes involving the ego. And finally, we could define the self in transcendental terms, as the witnessing self, which is usually addressed in contemplative practices. These selves may be seen separately and in relation to each other. Thus, including an embodied approach and a system lens to individual functioning and wellbeing, along with the exploration of key elements permeating different dimensions of wellbeing can offer a potential contribution to wellbeing models.

## Limitations

Participants included in this study were self-selected, and thus were likely to represent practitioners who were highly motivated and interested in providing their insights on yoga and wellbeing. Similar to previous research on yoga practitioners (e.g., Kishida et al., 2018; Cartwright et al., 2020), participants were mostly highly educated and middle-aged females who identified as Caucasian living in Westernized countries. These factors need to be taken into consideration as they might have influenced to some extent the content of responses and themes we identified.

Due to the methodology used in this study, we cannot and do not claim causality between the AY practice or any aspect of the practice and the themes that emerged within each dimension of wellbeing. Similarly, we cannot and do not claim that these themes are exclusive to regular AY practitioners. While the themes within each dimension may be unique to this sample, some overlaps may exist with other populations. Our study did not focus on the comparison of our group of practitioners with other groups. However, identifying what arose from their perspectives might give insights into dimensions that could be relevant for other populations as well, and could be considered in future research.

Finally, we reiterate our acknowledgement that our analysis and generation of the emergent model represent one way of organizing, interpreting, and integrating information provided by a particular group of people in a specific timepoint, according to our backgrounds, experiences, and expertise. Although these elements may be considered a limitation, they also allowed us to synthesize complex information using an interdisciplinary perspective to provide, what we believe, is the most appropriate and cohesive framework for this case.

## Conclusion

Conceptualizations of regular AYPs about the physical, emotional, psychological, social, and spiritual dimensions of wellbeing unearthed elements supporting the current wellbeing literature. Other elements going beyond mainstream subjective wellbeing frameworks were also revealed and interpreted through additional theoretical perspectives. Whilst providing a definition for each dimension can be considered a relevant contribution in and of itself, further analysis of the five dimensions allowed us to decipher connections within each and amongst the five dimensions. This meta-reflexive analysis offered a multileveled view of the self in relation to wellbeing, as a system operating at a foundational, optimal functioning, and contemplative and transcendental level. Shared meanings across dimensions and within each level signify different ways of knowing about wellbeing, whether it is about the absence and management of suffering, personal development and growth, or meta-awareness across planes of existence.

Findings from this study and the resulting preliminary model point to the relevance of understanding wellbeing according to different levels in which the self-functions and engages with themselves, with others, the world, and beyond. This translates into grappling with complexity and integrating multiple disciplines and lenses in wellbeing theory and research, rather than reducing the construct to isolated parts or to only a few components. This implies the use of alternate paradigms and the development of integrative models to understand how mind and body can work together towards wellbeing.

## Data availability statement

The raw data supporting the conclusions of this article will be made available by the authors, without undue reservation.

## Ethics statement

The studies involving human participants were reviewed and approved by University of Melbourne’s Human Research Ethics Committee (protocol #1955377.1). The patients/participants provided their written informed consent to participate in this study.

## Author contributions

DR-D is the lead author, designed and planned research design, conducted reflexive thematic analysis, reported and discussed findings, and generated the manuscript. HS and MK are secondary authors, collaborated in research design and planning, provided reflexive feedback on analysis, and revised and edited the manuscript. All authors contributed to the article and approved the submitted version.

## Funding

This research is part of a PhD thesis that has been supported by the National Agency for Research and Development (ANID) through Becas Chile (grant no. 72190189).

## Conflict of interest

The authors declare that the research was conducted in the absence of any commercial or financial relationships that could be construed as a potential conflict of interest.

## Publisher’s note

All claims expressed in this article are solely those of the authors and do not necessarily represent those of their affiliated organizations, or those of the publisher, the editors and the reviewers. Any product that may be evaluated in this article, or claim that may be made by its manufacturer, is not guaranteed or endorsed by the publisher.

## Footnotes

^1^ https://www.qualtrics.com/au/
