# Supplementary material for: Going within, between and beyond: An exploration of regular Ashtanga Yoga practitioners’ conceptualizations of five dimensions of wellbeing
Source: Front Psychol. 2022 Dec 21;13:1018620. doi: 10.3389/fpsyg.2022.1018620 (PMC9811318; doi:10.3389/fpsyg.2022.1018620)
Supplement: Supplementary file 1 [file Data_Sheet_1.docx]

**Supplement 1.** *Questions Included from the Ashtanga Yoga and Wellbeing Online Survey*

| Topic | Item | Type |
| --- | --- | --- |
| Ashtanga Yoga Practice | | |
| Criterion for selection | Do you consider yourself a regular Ashtanga Yoga practitioner? | Close-ended |
| Years of AY practice | How many years have you been practising Ashtanga Yoga? | Close-ended |
| Years of regular AY practice | How long have you been practising Ashtanga Yoga on a regular basis? | Close-ended |
| AY practice modality | What is your Ashtanga Yoga practice like? Select all that apply | Close-ended |
| Frequency | How many days do you usually practice per week? | Close-ended |
| Duration | How many minutes does your practice usually last, during an average session? | Close-ended |
| Time of the day | At what time do you usually practice? | Close-ended |
| Consistency | In a scale from 0 to 10, with 0 being completely inconsistent and 10 being completely consistent, how consistent is your practice? | Close-ended |
| Asana sequence | What sequence(s) are you currently practising? Select the option that best represents your regular asana practice | Close-ended |
| AY elements | What elements does your Ashtanga Yoga practice include? Select all that apply | Close-ended |
| Ashtanga Yoga Philosophy | | |
| Introduction to questions on eight limbs | Ashtanga Yoga can be translated as the “8 limbs of yoga” and refer not only to the physical practice of yoga, but also to a philosophical framework describing 8 different and interconnected aspects of yoga.  These eight aspects (i.e. yama, niyama, asana, pranayama, pratyahara, dharana, dhyana, samadhi) might be or not a part of your practice, and might vary in terms of their presence.   On a scale of 0 to 10, with 0 being never and 10 being always, please indicate to what extent you consider the presence of each element in your regular practice and/or life: | - |
| Yama | To what extent do you consider and apply moral observances (e.g. non-violence, truthfulness, non-stealing, non-temptation, non-collecting) in your behaviour with others? | Close-ended |
| Niyama | To what extent do you consider and apply moral inner observances (e.g. cleanliness, contentment, self-discipline, self-study, devotion) in your own behaviour? | Close-ended |
| Asana | To what extent do you incorporate physical postures into your own practice? | Close-ended |
| Pranayama | To what extent do you practice yoga breathing techniques? | Close-ended |
| Pratyahara | To what extent do you practice withdrawal from your senses during your practice? | Close-ended |
| Dharana | To what extent do you experience a state of concentration during your practice? | Close-ended |
| Dhyana | To what extent do you experience a state of meditation during your practice? | Close-ended |
| Samadhi | To what extent do you experience a state of bliss, absorption and oneness in your practice? | Close-ended |
| Relevance of philosophy | On a scale from 0 to 10, 0 being completely irrelevant and 10 being completely relevant, how relevant is yoga philosophy to your own practice? | Close-ended |
| Philosophy frequency | How often do you engage with yoga philosophy? | Close-ended |
| Type of yoga philosophy | In which type(s) of yoga philosophy activity do you engage with? Select all that apply | Close-ended |
| Wellbeing Dimensions | | |
| Introduction to wellbeing dimensions | Wellbeing is often defined as comprising of different areas, including but not limited to physical, emotional, psychological, social and spiritual. How would you define each of these dimensions? |  |
| Physical dimension | Physical wellbeing | Open-ended |
| Emotional dimension | Emotional wellbeing | Open-ended |
| Psychological dimension | Psychological wellbeing | Open-ended |
| Social dimension | Social wellbeing | Open-ended |
| Spiritual dimension | Spiritual wellbeing | Open-ended |
| Other dimensions | Any additional area that you consider important and has not been included?  Please comment in the box below which area/s and how you would define it/them: | Open-ended |
| Questions about the relation between AY practice and wellbeing | In a scale from 0 to 10, with 0 being not at all and 10 being completely influenced, to what extent each of the following areas of wellbeing have been positively influenced by your yoga practice? |  |
|  | Physical | Close-ended |
|  | Emotional | Close-ended |
|  | Psychological | Close-ended |
|  | Social | Close-ended |
|  | Spiritual | Close-ended |
| Demographics | | |
| Age | What is your age? | Close-ended |
| Gender | What is your gender? | Close-ended |
| Location | Where do you live? | Close-ended |
| Area | In which are do you live? | Close-ended |
| Citizenship | What is your nationality? | Close-ended |
| Ethnicity | Please specify your ethnicity | Close-ended |
| Education | What is the highest degree or level of education you have completed? | Close-ended |
| Spiritual tradition | With which of the following spiritual or religious traditions do you relate the most? | Close-ended |
|  | Comments | Open-ended |

**Supplement 2.** *Demographic characteristics of the current sample of regular AY practitioners versus the broader set of surveys completed.*

| Characteristic | Category | Regular AY Practitioners  N=137 | | | | Broader Sample  N=352 | | | | |
| --- | --- | --- | --- | --- | --- | --- | --- | --- | --- | --- |
|  |  | N | % | N | | | % | |  |  |
| Gender | Female  Male  Other/Not reported | 108  25  4 | 78.83%  18.25%  2.92% | 253  61  38 | | | 71.88%  17.33%  10.79% | |  |  |
| Age | 18 to 24 years old  25 to 34 years old  35 to 44 years old  45 to 54 years old  55 to 64 years old  65 to 74 years old  75 years old and above  Not reported | 11  44  39  29  12  1  1  0 | 8.03%  32.12%  28.47%  21.17%  8.76%  0.73%  0.73%  0.00% | 25  108  94  69  24  1  1  30 | | | 7.10%  30.68%  26.70%  19.60%  6.82%  0.28%  0.28%  8.52% | |  |  |
| Location | Americas  Europe  Asia  Oceania  Africa  Not reported | 50  32  12  37  2  4 | 36.50%  23.36%  8.76%  27.01%  1.46%  2.92% | 120  88  36  69  2  37 | | | 30.11%  23.58%  6.25%  19.60%  0.57%  10.51% | |  |  |
| Area | Urban  Sub-urban  Rural  Other/Not reported | 84  37  14  2 | 61.31%  27.01%  10.22%  1.46% | 204  79  32  37 | | | 57.95%  22.44%  9.09%  10.51% | |  |  |
| Citizenship | Same as location  Different from location  Not reported | 102  30  5 | 74.45%  21.90%  3.65% | 250  70  32 | | | 71.02%  19.89%  9.09% | |  |  |
| Education | High school education incomplete  High school education complete  Trade vocational  Bachelor  Master  Doctorate  Not reported | 0  7  11  60  45  14  0 | 0.00%  5.11%  8.03%  43.80%  32.85%  10.22%  0.00% | 6  16  24  131  110  33  32 | | | 1.70%  4.55%  6.82%  37.22%  31.25%  9.38%  9.09% | |  |  |
| Survey Language | English  Spanish | 116  21 | 84.67%  15.33% | 302  50 | | | 85.80%  14.20% | |  |  |
| Ethnicity | Arabic  African  Asian  Caucasian  Hispanic or Latino  Native American  Multiethnic  Other/Not reported | 0  1  14  76  19  5  13  9 | 0.00%  0.73%  10.22%  55.47%  13.87%  3.65%  9.49%  5.84% | 1  2  33  182  48  8  29  49 | | | 0.28%  0.57%  9.38%  51.70%  13.64%  2.27%  8.24%  13.92% | |  |  |
| Spiritual Tradition | Buddhism  Christianity  Hinduism  Islam  Judaism  Atheism  Agnosticism  Aboriginal Spirituality  None of the above  Other/Not reported | 26  23  7  1  1  16  12  1  42  8 | 18.98%  16.79%  5.11%  0.73%  0.73%  11.68%  8.76%  0.73%  30.66%  5.84% | | 60  63  20  2  3  44  29  2  73  56 | | | 17.05%  17.90%  5.68%  0.57%  0.85%  12.50%  8.24%  0.57%  20.74%  15.91% | |  |

**Supplement 3.** *Characteristics of regular Ashtanga Yoga practitioners’ (RAYP) yoga practice versus the broader sample of participants*

| Aspect of AY practice | Category | RAYP  N=137 | | Broader Sample  N=352 | |
| --- | --- | --- | --- | --- | --- |
|  |  | N | % | N | % |
| Years of AY practice | Less than 3 months  3 to 6 months  6 months to 12 months  1 to 2 years  3 to 5 years  6 to 10 years  11 to 15 years  16 to 20 years  More than 20 years  Not reported | 1  2  6  25  45  21  17  16  4  0 | 0.73%  1.46%  4.38%  18.25%  32.85%  15.33%  12.41%  11.68%  2.92%  0.00% | 14  9  21  53  84  52  27  27  11  54 | 3.98%  2.56%  5.97%  15.06%  23.86%  14.77%  7.67%  7.67%  3.13%  15.34% |
| Years of regular AY practice | Less than 3 months  3 to 6 months  6 months to 12 months  1 to 2 years  3 to 5 years  6 to 10 years  11 to 15 years  16 to 20 years  More than 20 years  Not reported | 3  7  10  26  40  26  10  11  3  1 | 2.19%  5.11%  7.30%  18.98%  29.20%  18.98%  7.30%  8.03%  2.19%  0.73% | 4  12  21  47  66  44  16  19  4  119 | 1.14%  3.41%  5.97%  13.35%  18.75%  12.50%  4.55%  5.40%  1.14%  33.81% |
| AY practice modality | Mysore style  Traditional Sanskrit-led class  Led-class (primary or intermediate)  Guided AY based class (different each class)  Self-practice  Mysore and traditional Sanskrit-led class  Mysore and led class  Mysore and guided AY based class  Mysore, Sanskrit-led and led-class  Mysore, Sanskrit-led and guided AY based  Mysore, Sanskrit-led, led-class and guided  Mysore, led-class and guided AY based  Mysore and Self-practice  Sanskrit-led and led class  Sanskrit-led and guided AY based class  Led-class and guided AY based class  Sanskrit-led, led-class and guided AY based  Other  Not reported | 64  1  2  4  3  34  9  1  11  0  3  2  1  0  0  0  1  1  0 | 46.72%  0.73%  1.46%  2.92%  2.19%  24.82%  6.57%  0.73%  8.03%  0.00%  2.19%  1.46%  0.73%  0.00%  0.00%  0.00%  0.73%  0.73%  0.00% | 122  7  8  13  3  69  18  2  27  3  7  8  1  2  1  1  2  3  55 | 34.66%  1.99%  2.27%  3.69%  0.85%  19.60%  5.11%  0.57%  7.67%  0.85%  1.99%  2.27%  0.28%  0.57%  0.28%  0.28%  0.57%  0.85%  15.63% |
| Frequency | Less than once a week  1 to 2 days per week  3 to 4 days per week  5 to 6 days per week  Everyday  It depends  Not reported | 0  10  40  75  7  4  1 | 0.00%  7.30%  29.20%  54.74%  5.11%  2.92%  0.73% | 5  38  78  135  13  27  56 | 1.42%  10.80%  22.16%  38.35%  3.69%  7.67%  15.91% |
| Duration | Less than 20 minutes  20 to 40 minutes  41 to 60 minutes  61 to 80 minutes  81 to 100 minutes  101 to 120 minutes  More than 120 minutes  It depends  Not reported | 0  1  16  46  33  30  10  1  0 | 0.00%  0.73%  11.68%  33.58%  24.09%  21.90%  7.30%  0.73%  0.00% | 4  8  39  90  83  52  17  5  54 | 1.14%  2.27%  11.08%  25.57%  23.58%  14.77%  4.83%  1.42%  15.34% |
| Time of the day | 6am to 10am  11am to 1pm  2pm to 5pm  6pm to 9pm  It depends  Not reported | 85  11  5  11  25  0 | 62.04%  8.03%  3.65%  8.03%  18.25%  0.00% | 178  20  12  44  44  54 | 50.57%  5.68%  3.41%  12.50%  12.50%  15.34% |
| Consistency | 1  2  3  4  5  6  7  8  9  10  Not reported | 0  0  0  3  2  12  32  39  26  21  2 | 0.00%  0.00%  0.00%  2.19%  1.46%  8.76%  23.36%  28.47%  18.98%  15.33%  1.46% | 3  6  9  11  15  32  64  74  43  35  60 | 0.85%  1.70%  2.56%  3.13%  4.26%  9.09%  18.18%  21.02%  12.22%  9.94%  17.05% |
| Asana sequence | Surya Namaskar A and B  Surya Namaskar and standing  Primary series up to Janu C  Primary series up to Navasana  Second half of Primary series  Full Primary series  Primary up to 1^st^ half of Intermediate  Primary up to 2^nd^ half of Intermediate  Full Primary and Intermediate  Primary, Int. up to 1^st^ half of Adv. A  Primary, Int. up to 2^nd^ half of Adv. A  Full Primary, Int. and Adv. A  Full Primary, Int. and Adv. A and B  Other  Not reported | 0  1  3  20  5  37  45  7  9  5  1  1  2  1  0 | 0.00%  0.73%  2.19%  14.60%  3.65%  27.01%  32.85%  5.11%  6.57%  3.65%  0.73%  0.73%  1.46%  0.73%  0.00% | 6  4  6  54  9  81  81  10  15  8  1  3  2  16  56 | 1.70%  1.14%  1.70%  15.34%  2.56%  23.01%  23.01%  2.84%  4.26%  2.27%  0.28%  0.85%  0.57%  4.55%  15.91% |
| AY elements | Free breathing with sound  Use of bandhas  Use of drishti  Chanting opening and closing mantras  Meditation before or after practice  Pranayama before or after practice  Resting on moon days  Vinyasa throughout practice  Other  Not reported | 123  126  130  121  66  71  93  133  9  0 | 89.78%  91.97%  94.89%  88.32%  48.18%  51.82%  67.88%  97.08%  6.57%  0.00% | 253  251  266  244  139  124  190  276  18  54 | 71.88%  71.31%  75.57%  69.32%  39.49%  35.23%  53.98%  78.41%  5.11%  15.34% |

**Supplement 4.** *Regular* *AYPs’ engagement in Ashtanga Yoga Philosophy*

| Aspect of Yoga Philosophy | Category | RAYP  N=137 | | | Broader Sample  N=352 | |
| --- | --- | --- | --- | --- | --- | --- |
|  |  | N | % | N | | % |
| Yama | 0  1  2  3  4  5  6  7  8  9  10  Not reported | 0  0  0  1  1  8  5  36  47  24  15  0 | 0.00%  0.00%  0.00%  0.73%  0.73%  5.84%  3.65%  26.28%  34.31%  17.52%  10.95%  0.00% | 1  2  2  3  2  22  17  63  79  46  31  84 | | 0.28%  0.57%  0.57%  0.85%  0.57%  6.25%  4.83%  17.90%  22.44%  13.07%  8.81%  23.86% |
| Niyama | 0  1  2  3  4  5  6  7  8  9  10  Not reported | 0  0  1  1  5  8  16  31  48  17  10  0 | 0.00%  0.00%  0.73%  0.73%  3.65%  5.84%  11.68%  22.63%  35.04%  12.41%  7.30%  0.00% | 1  0  2  4  12  19  34  67  77  31  18  87 | | 0.28%  0.00%  0.57%  1.14%  3.41%  5.40%  9.66%  19.03%  21.88%  8.81%  5.11%  24.72% |
| Asana | 0  1  2  3  4  5  6  7  8  9  10  Not reported | 0  0  0  0  1  5  0  8  25  27  70  1 | 0.00%  0.00%  0.00%  0.00%  0.73%  3.65%  0.00%  5.84%  18.25%  19.71%  51.09%  0.73% | 0  1  0  1  3  12  6  21  46  56  120  86 | | 0.00%  0.28%  0.00%  0.28%  0.85%  3.41%  1.70%  5.97%  13.07%  15.91%  34.09%  24.43% |
| Pranayama | 0  1  2  3  4  5  6  7  8  9  10  Not reported | 0  1  1  3  1  6  9  14  31  27  44  0 | 0.00%  0.73%  0.73%  2.19%  0.73%  4.38%  6.57%  10.22%  22.63%  19.71%  32.12%  0.00% | 1  2  3  6  6  19  18  25  64  47  77  84 | | 0.28%  0.57%  0.85%  1.70%  1.70%  5.40%  5.11%  7.10%  18.18%  13.35%  21.88%  23.86% |
| Pratyahara | 0  1  2  3  4  5  6  7  8  9  10  Not reported | 2  3  6  8  8  21  15  32  22  11  7  2 | 1.46%  2.19%  4.38%  5.84%  5.84%  15.33%  10.95%  23.36%  16.06%  8.03%  5.11%  1.46% | 6  7  17  13  20  37  35  50  41  21  12  93 | | 1.70%  1.99%  4.83%  3.69%  5.68%  10.51%  9.94%  14.20%  11.65%  5.97%  3.41%  26.42% |
| Dharana | 0  1  2  3  4  5  6  7  8  9  10  Not reported | 0  0  0  0  2  14  13  32  49  20  7  0 | 0.00%  0.00%  0.00%  0.00%  1.46%  10.22%  9.49%  23.36%  35.77%  14.60%  5.11%  0.00% | 0  2  1  3  7  24  33  58  83  36  18  87 | | 0.00%  0.57%  0.28%  0.85%  1.99%  6.82%  9.38%  16.48%  23.58%  10.23%  5.11%  24.72% |
| Dhyana | 0  1  2  3  4  5  6  7  8  9  10  Not reported | 1  2  3  4  5  15  22  35  29  15  6  0 | 0.73%  1.46%  2.19%  2.92%  3.65%  10.95%  16.06%  25.55%  21.17%  10.95%  4.38%  0.00% | 5  6  7  14  9  33  45  59  49  25  12  88 | | 1.42%  1.70%  1.99%  3.98%  2.57%  9.38%  12.78%  16.76%  13.92%  7.10%  3.41%  25.00% |
| Samadhi | 0  1  2  3  4  5  6  7  8  9  10  Not reported | 3  2  5  15  10  22  15  24  23  12  6  0 | 2.19%  1.46%  3.65%  10.95%  7.30%  16.06%  10.95%  17.52%  16.79%  8.76%  4.38%  0.00% | 5  10  12  26  24  33  31  38  51  20  13  89 | | 1.42%  1.70%  3.41%  7.39%  6.82%  9.38%  8.81%  10.80%  14.49%  5.68%  3.69%  25.28% |
| Relevance of philosophy | 0  1  2  3  4  5  6  7  8  9  10  Not reported | 0  0  1  4  4  7  14  18  26  12  50  1 | 0.00%  0.00%  0.73%  2.92%  2.92%  5.11%  10.22%  13.14%  18.98%  8.76%  36.50%  0.73% | 2  3  6  7  11  16  23  35  54  29  78  88 | | 0.57%  0.85%  1.70%  1.99%  3.13%  4.55%  6.53%  9.94%  15.34%  8.24%  22.16%  25.00% |
| Philosophy frequency | Never  Less than once per month  Once or twice per month  Once or twice per week  Almost daily  Not reported | 1  21  33  34  48  0 | 0.73%  15.33%  24.09%  24.82%  35.04%  0.00% | 12  44  58  70  83  85 | | 3.41%  12.50%  16.48%  19.89%  23.58%  24.15% |
| Type of yoga philosophy | After class conference by teacher  Study and chanting of mantras  Yoga teacher training programs  Yoga retreats  Public events  Face to face courses  Online courses  Reading books and texts  Online videos  Reading blogs  Reading social media posts  Other  Not reported | 53  69  57  46  26  38  39  114  71  73  74  10  1 | 38.69%  50.36%  41.61%  33.58%  18.98%  27.74%  28.47%  83.21%  51.82%  53.28%  54.01%  7.30%  0.73% | 97  119  80  73  43  61  63  208  124  125  126  18  100 | | 27.57%  33.81%  22.73%  20.74%  12.22%  17.33%  17.90%  59.09%  35.23%  35.51%  35.80%  5.11%  28.41% |

**Supplement 5.** *Perceived Influence of Yoga Practice on Different Dimensions of Regular AYPs’ Wellbeing*

| Wellbeing Dimension | Category | RAYP  N=137 | | | Broader Sample  N=352 | |
| --- | --- | --- | --- | --- | --- | --- |
|  |  | N | % | N | | % |
| Physical | 0  1  2  3  4  5  6  7  8  9  10  Not reported | 1  0  0  0  1  1  2  3  15  23  87  4 | 0.73%  0.00%  0.00%  0.00%  0.73%  0.73%  1.46%  2.19%  10.95%  16.79%  63.50%  2.92% | 1  1  1  0  1  3  4  10  23  36  109  163 | | 0.28%  0.28%  0.28%  0.00%  0.28%  0.85%  1.14%  2.84%  6.53%  10.23%  30.97%  46.31% |
| Emotional | 0  1  2  3  4  5  6  7  8  9  10  Not reported | 1  0  0  0  1  0  3  20  21  22  64  5 | 0.73%  0.00%  0.00%  0.00%  0.73%  0.00%  2.19%  14.60%  15.33%  16.06%  46.72%  3.65% | 1  1  0  0  1  1  6  23  35  31  87  166 | | 0.28%  0.28%  0.00%  0.00%  0.28%  0.28%  1.70%  6.53%  9.94%  8.81%  24.71%  47.16% |
| Psychological | 0  1  2  3  4  5  6  7  8  9  10  Not reported | 1  0  1  0  0  1  6  7  27  21  66  7 | 0.00%  0.00%  073%  0.00%  0.00%  0.73%  4.38%  5.11%  19.71%  15.33%  48.18%  5.11% | 1  2  1  0  1  2  8  11  39  31  87  169 | | 0.28%  0.57%  0.28%  0.00%  0.28%  0.57%  2.27%  3.13%  11.08%  8.81%  24.72%  48.01% |
| Social | 0  1  2  3  4  5  6  7  8  9  10  Not reported | 1  1  1  5  3  9  20  20  22  16  31  8 | 0.73%  0.73%  0.73%  3.65%  2.19%  6.57%  14.60%  14.60%  16.06%  11.68%  22.63%  5.84% | 1  2  5  6  6  14  26  28  34  17  43  170 | | 0.28%  0.57%  1.42%  1.70%  1.70%  3.98%  7.39%  7.95%  9.66%  4.83%  12.22%  48.30% |
| Spiritual | 0  1  2  3  4  5  6  7  8  9  10  Not reported | 1  0  0  3  1  3  4  9  22  18  66  10 | 0.73%  0.00%  0.00%  2.19%  0.73%  2.19%  2.92%  6.57%  16.06%  13.14%  48.18%  7.30% | 1  2  0  3  4  7  6  13  30  28  83  175 | | 0.28%  0.57%  0.00%  0.85%  1.14%  1.99%  1.70%  3.69%  8.52%  7.95%  23.58%  49.72% |

**Supplement 6.** *Perceived Relevance of Wellbeing Dimensions in Regular AYPs*

| Wellbeing Dimension | Category | RAYP  N=137 | | Broader Sample  N=352 | |
| --- | --- | --- | --- | --- | --- |
|  |  | N | % | N | % |
| Physical | 0  1  2  3  4  5  6  7  8  9  10  Not reported | 1  1  0  0  0  3  3  11  12  24  82  0 | 0.73%  0.73%  0.00%  0.00%  0.00%  2.19%  2.19%  8.03%  8.76%  17.52%  59.85%  0.00% | 2  1  0  0  0  3  3  13  29  30  117  154 | 0.57%  0.28%  0.00%  0.00%  0.00%  0.85%  0.85%  3.69%  8.24%  8.52%  33.24%  43.75% |
| Emotional | 0  1  2  3  4  5  6  7  8  9  10  Not reported | 0  0  0  0  0  2  4  5  17  35  74  0 | 0.00%  0.00%  0.00%  0.00%  0.00%  1.46%  2.92%  3.65%  12.41%  25.55%  54.01%  0.00% | 0  0  0  0  0  2  4  7  29  44  110  156 | 0.00%  0.00%  0.00%  0.00%  0.00%  0.57%  1.14%  1.99%  8.24%  12.50%  31.25%  44.32% |
| Psychological | 0  1  2  3  4  5  6  7  8  9  10  Not reported | 0  0  0  0  0  3  4  7  11  35  76  1 | 0.00%  0.00%  0.00%  0.00%  0.00%  2.19%  2.92%  5.11%  8.03%  25.55%  55.47%  0.73% | 0  0  0  0  0  3  5  10  23  44  110  157 | 0.00%  0.00%  0.00%  0.00%  0.00%  0.85%  1.42%  2.84%  6.53%  12.50%  31.25%  44.60% |
| Social | 0  1  2  3  4  5  6  7  8  9  10  Not reported | 1  1  0  1  2  6  9  13  23  21  60  0 | 0.73%  0.73%  0.00%  0.73%  1.46%  4.38%  6.57%  9.49%  16.79%  15.33%  43.80%  0.00% | 1  1  0  1  3  10  11  20  38  28  82  157 | 0.28%  0.28%  0.00%  0.28%  0.85%  2.84%  3.13%  5.68%  10.80%  7.95%  23.30%  44.60% |
| Spiritual | 0  1  2  3  4  5  6  7  8  9  10  Not reported | 1  3  0  1  0  4  11  10  12  22  68  5 | 0.73%  2.19%  0.00%  0.73%  0.00%  2.92%  8.03%  7.30%  8.76%  16.06%  49.64%  3.65% | 2  3  1  2  1  8  17  17  18  26  94  163 | 0.57%  0.85%  0.28%  0.57%  0.28%  2.27%  4.83%  4.83%  5.11%  7.39%  26.70%  46.31% |

**Supplement 7.** *Perceived Levels of Wellbeing Dimensions in Regular AYPs*

| Wellbeing Dimension | Category | RAYP  N=137 | | | Broader Sample  N=352 | |
| --- | --- | --- | --- | --- | --- | --- |
|  |  | N | % | N | | % |
| Physical | 0  1  2  3  4  5  6  7  8  9  10  Not reported | 0  0  0  0  1  2  6  28  40  39  18  3 | 0.00%  0.00%  0.00%  0.00%  0.73%  1.46%  4.38%  20.44%  29.20%  28.47%  13.14%  2.19% | 1  0  0  2  2  6  12  36  56  47  27  163 | | 0.28%  0.00%  0.00%  0.57%  0.57%  1.70%  3.41%  10.23%  15.91%  13.35%  7.67%  46.31% |
| Emotional | 0  1  2  3  4  5  6  7  8  9  10  Not reported | 0  0  1  2  1  10  9  37  43  22  10  2 | 0.00%  0.00%  0.73%  1.46%  0.73%  7.30%  6.57%  27.01%  31.39%  16.06%  7.30%  1.46% | 0  0  1  2  2  15  16  56  54  25  18  163 | | 0.00%  0.00%  0.28%  0.57%  0.57%  4.26%  4.55%  15.91%  15.34%  7.10%  5.11%  46.31% |
| Psychological | 0  1  2  3  4  5  6  7  8  9  10  Not reported | 0  0  1  2  3  10  12  36  37  19  12  5 | 0.00%  0.00%  0.73%  1.46%  2.19%  7.30%  8.76%  26.28%  27.01%  13.87%  8.76%  3.65% | 0  0  1  2  5  14  19  48  50  25  22  166 | | 0.00%  0.00%  0.28%  0.57%  1.42%  3.98%  5.40%  13.64%  14.20%  7.10%  6.25%  47.16% |
| Social | 0  1  2  3  4  5  6  7  8  9  10  Not reported | 1  1  2  1  5  17  16  26  30  22  11  5 | 0.73%  0.73%  1.46%  0.73%  3.65%  12.41%  11.68%  18.98%  21.90%  16.06%  8.03%  3.65% | 2  1  2  5  6  24  23  34  45  23  19  168 | | 0.57%  0.28%  0.57%  1.42%  1.70%  6.82%  6.53%  9.66%  12.78%  6.53%  5.40%  47.73% |
| Spiritual | 0  1  2  3  4  5  6  7  8  9  10  Not reported | 0  0  0  2  3  10  19  22  29  22  21  9 | 0.00%  0.00%  0.00%  1.46%  2.19%  7.30%  13.87%  16.06%  21.17%  16.06%  15.33%  6.57% | 1  0  0  2  5  17  26  36  37  25  31  172 | | 0.28%  0.00%  0.00%  0.57%  1.42%  4.83%  7.39%  10.23%  10.51%  7.10%  8.81%  48.86% |

**Supplement 8.** *Word Frequency of Words Included in Physical Dimension of Wellbeing Word Cloud*

| Word | Length | Count | Weighted Percentage |
| --- | --- | --- | --- |
| body | 4 | 73 | 10.91% |
| healthy | 7 | 37 | 5.53% |
| feeling | 7 | 23 | 3.44% |
| pain | 4 | 23 | 3.44% |
| physical | 8 | 22 | 3.29% |
| strong | 6 | 19 | 2.84% |
| free | 4 | 15 | 2.24% |
| good | 4 | 15 | 2.24% |
| able | 4 | 14 | 2.09% |
| health | 6 | 12 | 1.79% |
| fit | 3 | 10 | 1.49% |
| well | 4 | 10 | 1.49% |
| illness | 7 | 8 | 1.20% |
| physically | 10 | 8 | 1.20% |
| flexible | 8 | 7 | 1.05% |
| functioning | 11 | 6 | 0.90% |
| without | 7 | 6 | 0.90% |
| energetic | 9 | 5 | 0.75% |
| flexibility | 11 | 5 | 0.75% |
| movement | 8 | 5 | 0.75% |
| strength | 8 | 5 | 0.75% |
| activities | 10 | 4 | 0.60% |
| capable | 7 | 4 | 0.60% |
| digestion | 9 | 4 | 0.60% |
| disease | 7 | 4 | 0.60% |
| energy | 6 | 4 | 0.60% |
| enjoy | 5 | 4 | 0.60% |
| functions | 9 | 4 | 0.60% |
| injury | 6 | 4 | 0.60% |
| need | 4 | 4 | 0.60% |
| perform | 7 | 4 | 0.60% |
| sleep | 5 | 4 | 0.60% |
| tasks | 5 | 4 | 0.60% |
| things | 6 | 4 | 0.60% |
| want | 4 | 4 | 0.60% |
| ability | 7 | 3 | 0.45% |
| absence | 7 | 3 | 0.45% |
| activity | 8 | 3 | 0.45% |
| balance | 7 | 3 | 0.45% |
| best | 4 | 3 | 0.45% |
| conditions | 10 | 3 | 0.45% |
| daily | 5 | 3 | 0.45% |
| ease | 4 | 3 | 0.45% |
| enough | 6 | 3 | 0.45% |
| fitness | 7 | 3 | 0.45% |
| keeping | 7 | 3 | 0.45% |
| life | 4 | 3 | 0.45% |
| relaxed | 7 | 3 | 0.45% |
| self | 4 | 3 | 0.45% |
| active | 6 | 2 | 0.30% |

**Supplement 9.** *Word Frequency of Words Included in Emotional Dimension of Wellbeing Word Cloud*

| Word | Length | Count | Weighted Percentage |
| --- | --- | --- | --- |
| emotions | 8 | 46 | 6.91% |
| feeling | 7 | 26 | 3.90% |
| able | 4 | 17 | 2.55% |
| emotional | 9 | 16 | 2.40% |
| feel | 4 | 9 | 1.35% |
| good | 4 | 9 | 1.35% |
| control | 7 | 8 | 1.20% |
| others | 6 | 8 | 1.20% |
| balance | 7 | 7 | 1.05% |
| content | 7 | 7 | 1.05% |
| happy | 5 | 7 | 1.05% |
| peace | 5 | 7 | 1.05% |
| balanced | 8 | 6 | 0.90% |
| equanimity | 10 | 6 | 0.90% |
| loved | 5 | 6 | 0.90% |
| self | 4 | 6 | 0.90% |
| understand | 10 | 6 | 0.90% |
| without | 7 | 6 | 0.90% |
| calm | 4 | 5 | 0.75% |
| emotion | 7 | 5 | 0.75% |
| healthy | 7 | 5 | 0.75% |
| positive | 8 | 5 | 0.75% |
| stable | 6 | 5 | 0.75% |
| way | 3 | 5 | 0.75% |
| well | 4 | 5 | 0.75% |
| ability | 7 | 4 | 0.60% |
| accepting | 9 | 4 | 0.60% |
| aware | 5 | 4 | 0.60% |
| contentment | 11 | 4 | 0.60% |
| feelings | 8 | 4 | 0.60% |
| free | 4 | 4 | 0.60% |
| life | 4 | 4 | 0.60% |
| manage | 6 | 4 | 0.60% |
| mind | 4 | 4 | 0.60% |
| regulation | 10 | 4 | 0.60% |
| sense | 5 | 4 | 0.60% |
| situations | 10 | 4 | 0.60% |
| state | 5 | 4 | 0.60% |
| acceptance | 10 | 3 | 0.45% |
| appropriate | 11 | 3 | 0.45% |
| awareness | 9 | 3 | 0.45% |
| capacity | 8 | 3 | 0.45% |
| come | 4 | 3 | 0.45% |
| happiness | 9 | 3 | 0.45% |
| harmony | 7 | 3 | 0.45% |
| knowing | 7 | 3 | 0.45% |
| love | 4 | 3 | 0.45% |
| negative | 8 | 3 | 0.45% |
| ones | 4 | 3 | 0.45% |
| pleasant | 8 | 3 | 0.45% |

**Supplement 10.** *Word Frequency of Words Included in Psychological Dimension of Wellbeing Word Cloud*

| Word | Length | Count | Weighted Percentage |
| --- | --- | --- | --- |
| able | 4 | 17 | 2.51% |
| mental | 6 | 17 | 2.51% |
| mind | 4 | 15 | 2.22% |
| feeling | 7 | 14 | 2.07% |
| thoughts | 8 | 13 | 1.92% |
| life | 4 | 11 | 1.63% |
| anxiety | 7 | 9 | 1.33% |
| emotional | 9 | 8 | 1.18% |
| self | 4 | 8 | 1.18% |
| well | 4 | 8 | 1.18% |
| good | 4 | 7 | 1.04% |
| one | 3 | 7 | 1.04% |
| others | 6 | 7 | 1.04% |
| psychological | 13 | 7 | 1.04% |
| ability | 7 | 6 | 0.89% |
| emotions | 8 | 6 | 0.89% |
| health | 6 | 6 | 0.89% |
| knowing | 7 | 6 | 0.89% |
| mentally | 8 | 6 | 0.89% |
| depression | 10 | 5 | 0.74% |
| free | 4 | 5 | 0.74% |
| stress | 6 | 5 | 0.74% |
| understand | 10 | 5 | 0.74% |
| understanding | 13 | 5 | 0.74% |
| healthy | 7 | 4 | 0.59% |
| making | 6 | 4 | 0.59% |
| managing | 8 | 4 | 0.59% |
| stability | 9 | 4 | 0.59% |
| world | 5 | 4 | 0.59% |
| awareness | 9 | 3 | 0.44% |
| balanced | 8 | 3 | 0.44% |
| calm | 4 | 3 | 0.44% |
| cognitive | 9 | 3 | 0.44% |
| deal | 4 | 3 | 0.44% |
| feel | 4 | 3 | 0.44% |
| focus | 5 | 3 | 0.44% |
| harmony | 7 | 3 | 0.44% |
| keep | 4 | 3 | 0.44% |
| observer | 8 | 3 | 0.44% |
| see | 3 | 3 | 0.44% |
| something | 9 | 3 | 0.44% |
| state | 5 | 3 | 0.44% |
| symptoms | 8 | 3 | 0.44% |
| think | 5 | 3 | 0.44% |
| without | 7 | 3 | 0.44% |
| accept | 6 | 2 | 0.30% |
| accepting | 9 | 2 | 0.30% |
| actions | 7 | 2 | 0.30% |
| active | 6 | 2 | 0.30% |
| always | 6 | 2 | 0.30% |

**Supplement 11.** *Word Frequency of Words Included in Social Dimension of Wellbeing Word Cloud*

| **Word** | **Length** | **Count** | **Weighted Percentage** |
| --- | --- | --- | --- |
| others | 6 | 29 | 4.66% |
| relationships | 13 | 28 | 4.50% |
| people | 6 | 21 | 3.38% |
| social | 6 | 21 | 3.38% |
| friends | 7 | 18 | 2.89% |
| healthy | 7 | 16 | 2.57% |
| around | 6 | 13 | 2.09% |
| community | 9 | 13 | 2.09% |
| feeling | 7 | 13 | 2.09% |
| able | 4 | 11 | 1.77% |
| good | 4 | 11 | 1.77% |
| connected | 9 | 9 | 1.45% |
| connection | 10 | 9 | 1.45% |
| support | 7 | 9 | 1.45% |
| family | 6 | 8 | 1.29% |
| love | 4 | 8 | 1.29% |
| interact | 8 | 6 | 0.96% |
| life | 4 | 6 | 0.96% |
| environment | 11 | 5 | 0.80% |
| feel | 4 | 5 | 0.80% |
| needs | 5 | 5 | 0.80% |
| ability | 7 | 4 | 0.64% |
| care | 4 | 4 | 0.64% |
| empathy | 7 | 4 | 0.64% |
| interactions | 12 | 4 | 0.64% |
| network | 7 | 4 | 0.64% |
| respect | 7 | 4 | 0.64% |
| self | 4 | 4 | 0.64% |
| supportive | 10 | 4 | 0.64% |
| accept | 6 | 3 | 0.48% |
| accepted | 8 | 3 | 0.48% |
| beings | 6 | 3 | 0.48% |
| circle | 6 | 3 | 0.48% |
| connect | 7 | 3 | 0.48% |
| covered | 7 | 3 | 0.48% |
| enjoy | 5 | 3 | 0.48% |
| harmony | 7 | 3 | 0.48% |
| meaningful | 10 | 3 | 0.48% |
| sense | 5 | 3 | 0.48% |
| society | 7 | 3 | 0.48% |
| time | 4 | 3 | 0.48% |
| understand | 10 | 3 | 0.48% |
| alone | 5 | 2 | 0.32% |
| based | 5 | 2 | 0.32% |
| basic | 5 | 2 | 0.32% |
| boundaries | 10 | 2 | 0.32% |
| comfortable | 11 | 2 | 0.32% |
| communication | 13 | 2 | 0.32% |
| company | 7 | 2 | 0.32% |
| confidence | 10 | 2 | 0.32% |

**Supplement 12.** *Word Frequency of Words Included in Spiritual Dimension of Wellbeing Word Cloud*

| **Word** | **Length** | **Count** | **Weighted Percentage** |
| --- | --- | --- | --- |
| connection | 10 | 20 | 3.26% |
| feeling | 7 | 19 | 3.10% |
| life | 4 | 17 | 2.77% |
| connected | 9 | 14 | 2.28% |
| purpose | 7 | 12 | 1.96% |
| sense | 5 | 12 | 1.96% |
| self | 4 | 10 | 1.63% |
| higher | 6 | 9 | 1.47% |
| something | 9 | 8 | 1.31% |
| greater | 7 | 7 | 1.14% |
| meaning | 7 | 7 | 1.14% |
| nature | 6 | 7 | 1.14% |
| one | 3 | 7 | 1.14% |
| spiritual | 9 | 7 | 1.14% |
| universe | 8 | 7 | 1.14% |
| awareness | 9 | 6 | 0.98% |
| faith | 5 | 6 | 0.98% |
| world | 5 | 6 | 0.98% |
| content | 7 | 5 | 0.82% |
| deeper | 6 | 5 | 0.82% |
| think | 5 | 5 | 0.82% |
| divine | 6 | 4 | 0.65% |
| feel | 4 | 4 | 0.65% |
| peace | 5 | 4 | 0.65% |
| physical | 8 | 4 | 0.65% |
| practice | 8 | 4 | 0.65% |
| spirit | 6 | 4 | 0.65% |
| within | 6 | 4 | 0.65% |
| beliefs | 7 | 3 | 0.49% |
| believe | 7 | 3 | 0.49% |
| calm | 4 | 3 | 0.49% |
| catholic | 8 | 3 | 0.49% |
| connect | 7 | 3 | 0.49% |
| energy | 6 | 3 | 0.49% |
| finding | 7 | 3 | 0.49% |
| god | 3 | 3 | 0.49% |
| harmony | 7 | 3 | 0.49% |
| know | 4 | 3 | 0.49% |
| knowing | 7 | 3 | 0.49% |
| living | 6 | 3 | 0.49% |
| meditation | 10 | 3 | 0.49% |
| oneness | 7 | 3 | 0.49% |
| others | 6 | 3 | 0.49% |
| place | 5 | 3 | 0.49% |
| soul | 4 | 3 | 0.49% |
| understand | 10 | 3 | 0.49% |
| understanding | 13 | 3 | 0.49% |
| well | 4 | 3 | 0.49% |
| able | 4 | 2 | 0.33% |
| accept | 6 | 2 | 0.33% |

**Supplement 13.** *Key Words In Context for “Able” and “Feeling” Represented in Word Trees*


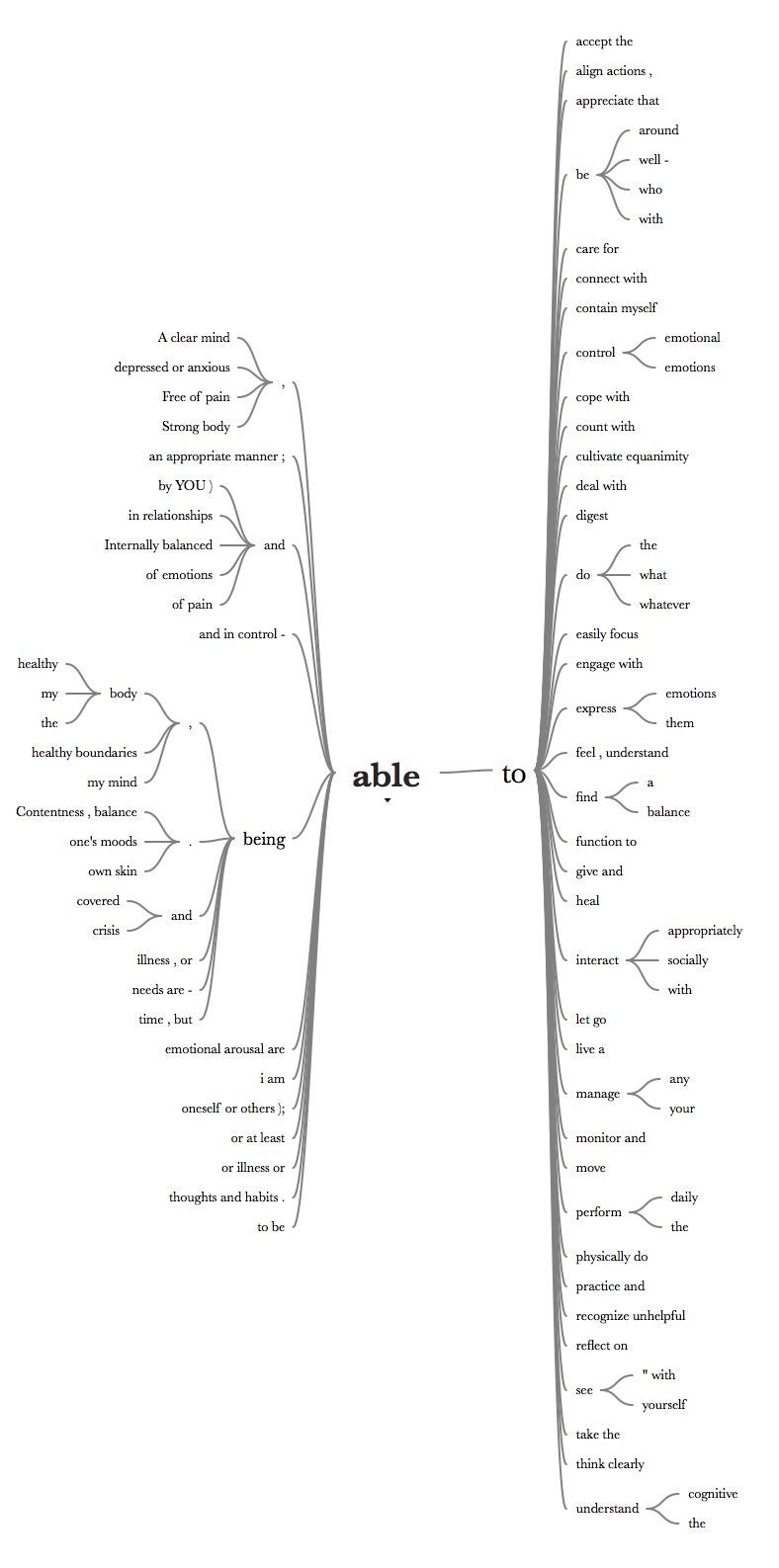


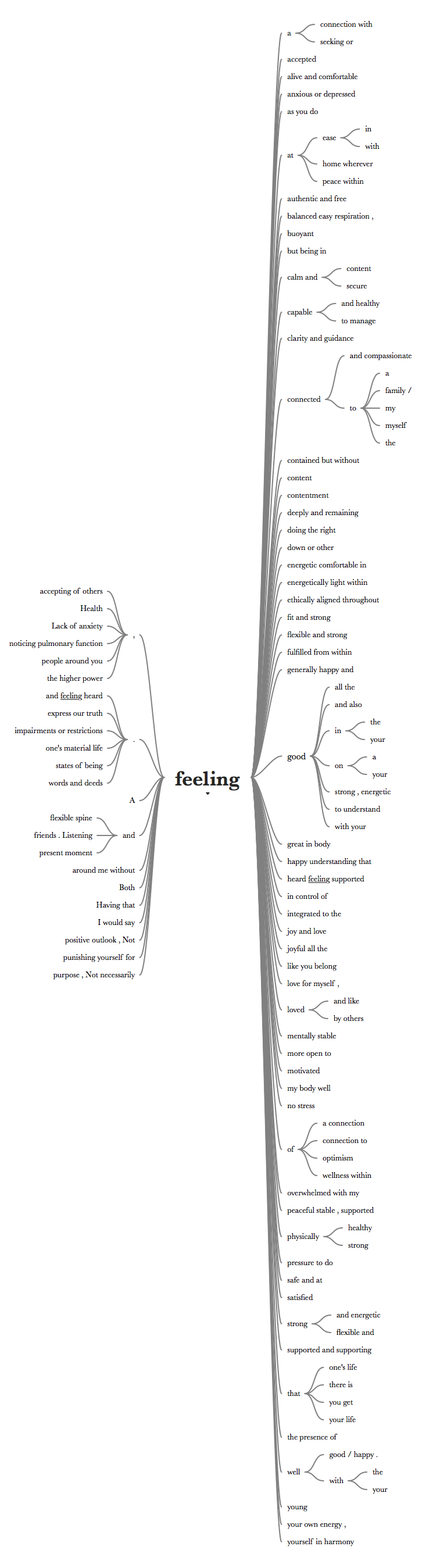


**Supplement 14.** *Summary of Themes for Each Dimension of Wellbeing*

| WB Dimensions | Themes | Sub-themes | N |
| --- | --- | --- | --- |
| Physical | *Being free from and able to manage physical suffering* | Freedom from illness | 25 |
|  |  | Longevity | 1 |
|  |  | Recovery | 1 |
|  | *Having a functioning body, responsive to internal and external demands* | A body capable of responding to demands | 12 |
|  |  | A body that functions well | 8 |
|  |  | A body with good mobility | 5 |
|  | *Having a sense of health and positive physicality* | Balance | 3 |
|  |  | Confidence | 1 |
|  |  | Endurance | 3 |
|  |  | Energy levels | 11 |
|  |  | Fitness | 10 |
|  |  | Flexibility | 6 |
|  |  | Health | 29 |
|  |  | Strength | 18 |
|  | *Consciously and actively inhabiting the body* | Agency and autonomy over the physical self | 12 |
|  |  | Contentment with the physical self | 2 |
|  |  | Healthy habits and choices | 18 |
|  |  | Physical awareness | 4 |
|  |  | Sense of embodied comfort or content | 8 |
| Emotional | *Being able to experience positive affect and the absence of negative affect* | Absence of negative affect | 5 |
|  |  | High intensity positive emotions | 7 |
|  |  | Low intensity positive emotions | 30 |
|  |  | Positive emotional states | 6 |
|  |  | Prosocial emotions | 9 |
|  | *Being able to embrace emotions, valuing negative affect and challenges* | Acceptance of pleasant and unpleasant emotions | 18 |
|  |  | Coping with challenges | 6 |
|  | *Having emotional awareness and regulation* | Emotional awareness | 14 |
|  |  | Emotional regulation | 37 |
|  | *Having emotional meta-awareness and an overall sense of balance and steadiness* | Emotional balance | 13 |
|  |  | Emotional meta-awareness | 18 |
|  |  | Emotional steadiness | 13 |
|  |  | Non-attachment to emotions | 9 |
| Psychological | *Being free from and being able to manage mental suffering* | Freedom from illness | 21 |
|  |  | Tools to cope with and manage illness | 9 |
|  | *Having a strong sense of identity and self* | Knowing and accepting oneself | 23 |
|  |  | Regulating oneself | 26 |
|  |  | Sense of control and ownership of oneself | 13 |
|  | *Experiencing a sense of mental steadiness* | Equanimity | 21 |
|  |  | Experiencing a sense of balance | 6 |
|  |  | Experiencing contentment | 8 |
|  |  | Mental focus and clarity | 8 |
|  |  | Mental stability | 15 |
|  | *Engaging in the contemplation of the self* | Having strategies to foster positive mental states | 16 |
|  |  | Meta-awareness of mental processes | 30 |
| Social | *Presence of social connections and social competence* | Existence of social relationships | 27 |
|  |  | Social competence | 11 |
|  | *Feeling connected and attuned to others and to the environment* | Feeling connected to others | 21 |
|  |  | Sense of community | 13 |
|  |  | Sense of social contribution | 4 |
|  |  | Social harmony | 4 |
|  | *Having high quality interpersonal relationships* | Acceptance | 10 |
|  |  | Authenticity | 8 |
|  |  | Capacity of enjoyment | 7 |
|  |  | Empathy and compassion | 7 |
|  |  | Feeling safe | 2 |
|  |  | Healthy, positive and meaningful relationships | 27 |
|  |  | Intimacy | 4 |
|  |  | Kindness | 4 |
|  |  | Reciprocity | 11 |
|  |  | Social support | 19 |
|  | *Engaging in the contemplation of the social self and relationships* | Acceptance of and contentment with social self and others | 12 |
|  |  | Balancing social life with solitude | 4 |
|  |  | Healthy social boundaries | 8 |
|  |  | Observance of social self and relationships | 11 |
| Spiritual | *A sense of connection to oneself and to an individual purpose and meaning in life* | Connection to self | 24 |
|  |  | Cultivating and experiencing positive states | 47 |
|  |  | Purpose and meaning | 10 |
|  | *A sense of connection to others and to the environment* | Connection to the environment | 15 |
|  |  | Connection to others | 13 |
|  | *Engaging in the contemplation of the self and connection to the transcendental self* | Connection to the higher self | 19 |
|  |  | Contemplation of the self and of human existence | 23 |
|  |  | Higher purpose and meaning | 10 |
|  | *A sense of connection to the divine and to a higher purpose and meaning* | Connection to the divine | 33 |
|  |  | Engaging in practices and applying teachings | 19 |
